# Substituting meat for mycoprotein reduces genotoxicity and increases the abundance of beneficial microbes in the gut: Mycomeat, a randomised crossover control trial

**DOI:** 10.1007/s00394-023-03088-x

**Published:** 2023-01-18

**Authors:** Dominic N. Farsi, Jose Lara Gallegos, Georgios Koutsidis, Andrew Nelson, Tim J. A. Finnigan, William Cheung, Jose L. Muñoz-Muñoz, Daniel M. Commane

**Affiliations:** 1 Department of Applied Sciences, University of Northumbria, Newcastle, UK; 2 Marlow Foods, Stokesley, UK

**Keywords:** Mycoprotein, Meat, Quorn, Genotoxicity, Microbiome, Colorectal cancer risk

## Abstract

**Purpose:**

The high-meat, low-fibre Western diet is strongly associated with colorectal cancer risk. Mycoprotein, produced from *Fusarium venanatum*, has been sold as a high-fibre alternative to meat for decades. Hitherto, the effects of mycoprotein in the human bowel have not been well considered. Here, we explored the effects of replacing a high red and processed meat intake with mycoprotein on markers of intestinal genotoxicity and gut health.

**Methods:**

Mycomeat (clinicaltrials.gov NCT03944421) was an investigator-blind, randomised, crossover dietary intervention trial. Twenty healthy male adults were randomised to consume 240 g day^−1^ red and processed meat for 2 weeks, with crossover to 2 weeks 240 g day^−1^ mycoprotein, separated by a 4-week washout period. Primary end points were faecal genotoxicity and genotoxins, while secondary end points comprised changes in gut microbiome composition and activity.

**Results:**

The meat diet increased faecal genotoxicity and nitroso compound excretion, whereas the weight-matched consumption of mycoprotein decreased faecal genotoxicity and nitroso compounds. In addition, meat intake increased the abundance of *Oscillobacter* and *Alistipes*, whereas mycoprotein consumption increased *Lactobacilli*, *Roseburia* and *Akkermansia*, as well as the excretion of short chain fatty acids.

**Conclusion:**

Replacing red and processed meat with the *Fusarium*-based meat alternative, mycoprotein, significantly reduces faecal genotoxicity and genotoxin excretion and increases the abundance of microbial genera with putative health benefits in the gut. This work demonstrates that mycoprotein may be a beneficial alternative to meat within the context of gut health and colorectal cancer prevention.

**Supplementary Information:**

The online version contains supplementary material available at 10.1007/s00394-023-03088-x.

## Introduction

Epidemiological data consistently associates red and processed meat consumption with an increased risk of colorectal cancer (CRC) [1–4]. Mechanistic studies reveal that meat consumption increases both faecal water genotoxicity and the excretion of faecal genotoxins, such as nitroso compounds (NOC) [5, 6]. Further, meat may displace plant foods, and thus fibre, from the diet, leading to a reduced production of anti-carcinogenic gut microbial metabolites such as butyrate [7].

This evidence is reflected in recommendations from both EatLancet and IARC to reduce meat consumption [8, 9]. However, reducing meat intake is challenging due to food preferences, social norms, and strongly held culinary traditions [10]. Meat alternatives may facilitate meat reduction without requiring drastic changes to culinary practice [11, 12]. Mycoprotein is a meat alternative produced from *Fusarium venenatum*, which is high in both protein and fibre [13]. From a gut health perspective, the fibre fraction is an interesting combination of 1,3/1,6 β-glucan, chitin and lesser studied mannoproteins [14]. In mixed-culture fermentations with faecal inoculate, the fibre fraction induces a significant increase in short chain fatty acids (SCFA) [15]. When compared to myofibrillar protein, the fermentation of mycoprotein yields lower concentrations of ammonia [16]. Previous work has shown cardiometabolic benefits when replacing meat with mycoprotein that may be attributable to its fibre composition [17, 18]. However, to date, there have been no human intervention studies of the effect of mycoprotein on CRC risk markers or other facets of gut health.

The objective of the present study (Mycomeat) was to compare the effects of substituting a high intake of red and processed meat with mycoprotein on biomarkers of CRC risk and gut health, namely, faecal water genotoxicity, faecal genotoxins, gut microbial composition, and the excreted metabolome.

## Methods

Procedures for this study were followed in accordance with the Declaration of Helsinki and were approved by the Northumbria University Ethics Committee (reference number 15274). All study participants provided written informed consent.

### Study design

**Fig. 1:**
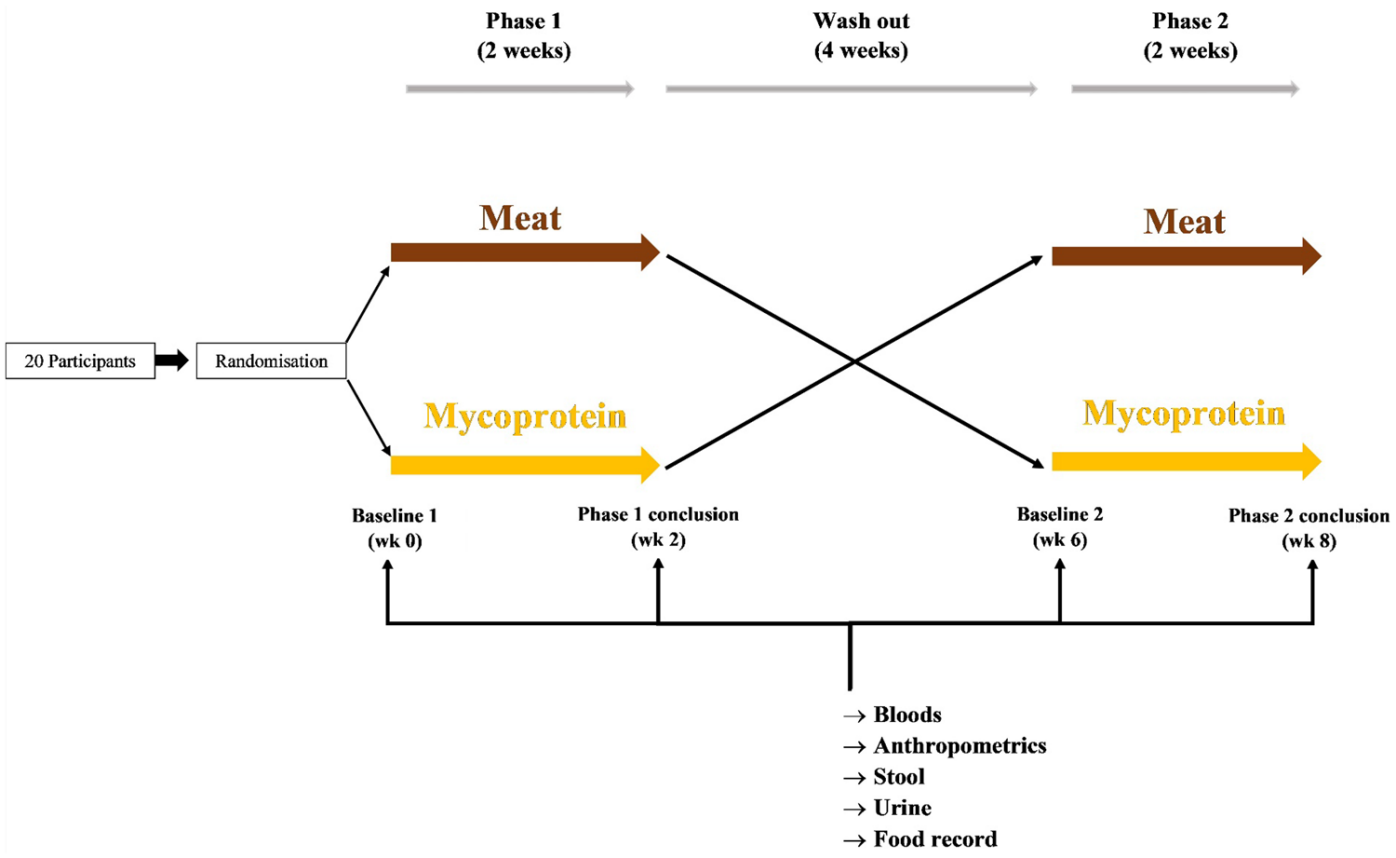
Mycomeat study design. Mycomeat was an investigator-blind randomised crossover design comprising two study phases (2 weeks) separated by a washout period (4 weeks). During the study phases, participants supplemented habitual diet with either 240 g/day of red and processed meat (Meat), or mycoprotein-based foods (Mycoprotein)

Mycomeat was a single-site, investigator-blind, randomised crossover trial (NCT03944421) among male adults randomised to consume either 240 g/day (uncooked weight) of red and processed meat (Meat) or mycoprotein, as Quorn™ products (Mycoprotein) for a 2-week period. Participants entered a 4-week washout period before swapping over to complete the alternative 2-week study arm (Fig. 1). The meat dose was selected to represent high intakes in the UK population and as a reflection of previous intervention trials assessing changes in faecal water genotoxicity in response to meat [6, 19]. The chronic feeding period of 2 weeks was selected based on this previous research, as well as a reflection of the average time taken to reach a new steady state for the microbiome in models of gut fermentation following a change in substrate [20]. A sample size of 20 to reach a statistical power of 80% at an *α*-level of 0.05 was established based on a repeated measures analysis of the primary end point, faecal water genotoxicity. This power calculation was informed by a previous report that found 300 g/day of red meat for 7 days increased faecal water genotoxicity compared to participants’ habitual diets (before intervention 9.6 ± 13.8, after intervention 16.7 ± 18.2) [21]. Participant recruitment was stopped at the completion of *n* = 20 participants.

### Participants

Participants were recruited from the North-East of England, UK, via on-campus poster advertisement and using a database of previous study participants. Inclusion criteria were: age 18–50 yr; BMI 18–30 kg/m^2^; willingness to refrain from pre- and probiotics, vitamin supplements as well as alcoholic beverages during the study. Exclusion criteria included: gastrointestinal disease; use of medications that affect gastrointestinal motility; use of antibiotic, prebiotic, or probiotics in the previous 3 months; use of tobacco or recreational drugs and history of coronary artery disease, diabetes, or other chronic disorders. We also excluded study participants who had been enrolled in dietary trials in the previous 3 months. All participants took part in a screening visit where they were characterised by blood parameters within standard clinical cutoff values to confirm study eligibility.

### Intervention

At the beginning of each study phase, pre-frozen study foods (14 × 240 g packs; equating to 240 g/day for 14 days) were provided to participants, with instructions for appropriate storage, preparation, and cooking. The study foods and instructions were packaged externally and transported in concealed boxes. The boxes were assigned A or B, depending on the study arm, with the participants randomly assigned to receive either A or B in the first study phase, followed by the alternative in the second phase. In this way, the investigators were blinded to diet order. Randomisation was carried out using an online sequence randomisation generator, with *n* = 12 randomised to Mycoprotein phase first and *n* = 8 randomised to Meat phase first. All mycoprotein products were supplied by Quorn™, and all meat products were sourced from Asda supermarket chain (UK), and both mycoprotein and meat products were distributed on-site at the research facility.

To promote compliance, products were provided based on a 7-day rotation and included the following: beef steak, pork sausages, ham slices, gammon steak, bacon, beef mince and hot dogs. For the Mycoprotein phase, the equivalent Quorn™ products were included: peppered steak, sausages, deli ham slices, gammon steak, bacon, mince, and hot dogs. During intervention periods, participants were asked to maintain their usual diet, but to avoid consuming any other meat or mycoprotein products other than the supplied study foods as well as additional high protein, fibre or probiotic supplements. As stated, the study foods were matched for weight (240 g uncooked); however, the total energy content of meat and mycoprotein products were closely matched to avoid introducing additional effects from energy intake between the diets. The nutritional composition of the study foods and differences in nutrients are included in Supplementary Material, Table1i and 1ii. 1-day food records were collected at baseline and during each study phase, from which energy and macronutrient intake were calculated using Nutritics nutrition analysis software (version 5.66 Education) [22].

### Sample collection

At baseline and conclusion of each study phase, participants visited the Brain, Performance and Nutrition Research Centre (BPNRC/NUTRAN, Northumbria University, Newcastle, UK) having fasted overnight (Fig. 1). At each visit, a first spot urine sample and stool sample (collected ≤ 10 h prior to study visit) were collected from participants. Urine samples were immediately put on ice, then aliquoted into sterile 1.5 ml Eppendorf tubes and stored at − 80 °C until analysis. Stool samples were put on ice at collection and then aliquoted into sterile 2 ml tubes and stored at − 80 °C for gut microbial analysis. The remainder of the sample was weighed, diluted 1:1 with PBS (Sigma) and homogenised in a stomacher (Seward Stomacher 400 Circulator) at 200 bts/min for 2 min. The homogenates were transferred into polypropylene tubes and centrifuged at 65,000 ×*g*, for 2 h, at 4 °C using an ultra-speed centrifuge system (Thermo Scientific Sorvall LYNX 6000 Superspeed Centrifuge). The supernatants were then filtered through a 0.44 µm polyethersulfone (PES) syringe filter prior to a second filtration through a 0.22 µm PES syringe filter (Fisher Scientific). The supernatants, representing the faecal water fraction, were aliquoted into sterile 1.5 ml Eppendorf tubes and stored at − 80 °C for analysis of faecal water genotoxicity and metabolites.

### Faecal water genotoxicity

The human colon adenocarcinoma cell line Caco-2 was used to test faecal water genotoxicity using a CometChip assay (Trevigen), a high-throughput version of the standard alkaline comet assay [23].

Caco-2 cells were purchased from the ECACC and used between passage 10 and 22. Then they were routinely cultured in Dulbecco’s modified Eagle medium (DMEM) supplemented with 0.1 mM of non-essential amino acids, 100 U/mL of penicillin, 0.1 g/mL of streptomycin, and 20% foetal bovine serum (FBS) (all Lonza UK). In the final passage prior to experimental procedures, the FBS was reduced to 10% of culture medium and experiments were carried out with this as carrier control.

The comet assay was performed using the CometChip protocol (Trevigen, USA). To ensure adequate blinding, an independent analyst prepared 10% (v/v) faecal extract in carrier media, prepared by passing the previously prepared faecal water through a 0.22 µm filter immediately prior to treating cells. 10% v/v faecal extract preparations were shown to be non-cytotoxic in a 24 h MTT assay (viability was maintained at over 90%) and thus this concentration was deemed appropriate for genotoxicity.

Caco-2 cells were split at 80% confluence, adjusted to 1 × 10^5^ cells per ml and loaded on to the CometChip in 100 μl aliquots. After allowing cells to embed, excess media was removed and 100 μl of (10% v/v) treatment added. After 30 min, the treatment media was removed, and the chip was washed with PBS and sealed in low melting point agarose. The comet assay was completed under alkali conditions. Sybr Gold stained comets were visualised under fluorescence (LEICA DM5000 Fluouresence Microscope) at 5× magnification. Image acquisition was completed by a second investigator with no knowledge of treatments.

Comet tails were assessed using the automated CometChip software (Trevigen, USA) which counted a minimum of 150 cell nuclei per well. Data represents a mean of four wells per treatment. 50 μM H_2_O_2_ and a carrier control were included as inter-assay controls. *T* test revealed a significant difference in DNA damage between inter-assay controls (*P* = 0.02).

### Faecal nitroso compounds

Faecal nitrates, nitrites and total NOC were determined using chemiluminescence as described previously [24]. To determine nitrite concentrations, 50 μl of faecal water was injected into a purge vessel containing 8 ml glacial acetic acid and 2 ml aqueous potassium iodide (50 mg/ml). Nitrogen was bubbled through a glass frit to mix the sample and transfer released nitric oxide to a Sievers NOA 280 analyser (Sievers, Boulder, CO, USA) via a condenser, an NaOH (1 mol/L) trap and a polypropylene filter (0.2 μm; Whatman, USA). The signal was processed using the instrument software. After every six injections, the purge vessel was emptied and refilled with fresh reagents. For quantification, known standards of sodium nitrite (1 to 10,000 nmol) were injected into the purge vessel filled with 8 ml glacial acetic acid and 2 ml aqueous potassium iodide (50 mg/ml).

For nitrate determination, the faecal water was incubated twofold with methanol for 30 min, followed by centrifugation at 14,000*g* at 4 °C for 5 min. 50 μl of the supernatant was injected into the purge vessel containing 8 ml vanadium (III) chloride solution (~ 0.4 g vanadium (III) chloride in 50 ml 1 M hydrochloric acid). The purge vessel was fitted with a water jacket to allow heating of the reagent to 96 °C and a cold water condenser (6 °C), using a circulating bath. Thereafter, the purge vessel was replenished with reagents as described above. The samples were quantified by comparing the area to the area of known standards of sodium nitrate (1 to 10,000 nmol). Results are expressed as nanomoles of total NOC.

### Microbial composition analysis by 16S ribosomal RNA amplicon sequencing

Stool aliquots were thawed prior to microbial DNA extraction using a Fast DNA Stool Mini Kit (Qiagen) according to the manufacturer’s instructions. 16S ribosomal RNA amplicon sequencing (rRNA) was performed at the NU-OMICS DNA sequencing research facility (Northumbria University). Sequencing libraries were prepared for targeted sequencing of the V4 region of the 16S rRNA gene from PCR amplicons as per the Schloss standard operating procedure (SOP) [25] using primers 515F and 806R [26]. Libraries were sequenced on the Illumina MiSeq platform (CA), using V2 (2 × 250), chemistry. Extraction kit and sequencing negative controls were prepared and sequenced simultaneously with all samples. Paired end reads were trimmed, merged, and processed by alignment to the SILVA database, followed by de novo clustering into operational taxonomic units (OTUs), and taxonomic assignment in Mothur, following the MiSeq SOP [25].

### Faecal volatile compounds

Automated headspace solid-phase microextraction (SPME) gas chromatography mass spectrometry (GC–MS) was conducted on an Agilent 7890A gas chromatograph, coupled to a CTC-PAL autosampler and a BencthTOF mass spectrometer (Markes Intl, Laitrisant, UK). Homogenised faecal samples (100–200 mg) were accurately weighed into 10 mL headspace vials, followed by the addition of 2 mL 26% NaCl and 5% metaphosphoric acid prior to sonication (5 min). The samples were then placed into the autosampler tray and incubated at 60 °C for 30 min, followed by a 30 s extraction onto a 75 μm Carboxen/ PDMS fibre (Supelco, Bellefonte, PA) and desorption in the GC injector set at 250 °C for 8 min. The injector was operated in the split mode at a split ratio of 20:1, using helium as a carrier gas at a constant flow of 1.15 mL/min. Chromatographic separation was achieved on VF-WaxMS ((L) 60 m × (D) 0.25 mm × (FT) 0.25 μm) capillary column (Agilent). The oven was held at 40 °C for 8 min followed by a temperature ramp at 4 °C/min to 100 °C, then 15 °C/min to 260 °C and held for 5 min. The mass spectrometer was operated in the total ion scan mode (m/z 30–450) with the ion source and transfer line temperatures held at 250 °C and 245 °C, respectively. The SCFA acetate, propionate, butyrate, valerate and hexanoate, branched chain fatty acids (BCFA) isobutyrate and isolvalerate, as well as cresol and phenol, were quantified using an external calibration curve.

### Metabolomics

Frozen aliquots of urine were thawed prior to normalisation through dilution with sterile distilled water to the lowest specific gravity measured by refractometry as previously described [27]. Following this, 225 μL of the sample was transferred to 9 mm glass screw thread vials (Thermo Scientific), followed by the addition of 75 μL acetonitrile and stored at − 80 °C until the time of analysis.

For faecal water, frozen aliquots of sample were thawed before centrifugation at 18,000*g* at 4 °C for 30 min. 225 μL of the supernatant was transferred to 9 mm glass screw thread vials (Thermo Scientific), followed by the addition of 75 μL acetonitrile and stored at − 80 °C until the time of analysis.

Hydrophilic liquid interaction chromatography (HILIC) metabolite profiling of the urine and faecal water samples was performed on a Vanquish Liquid Chromatography chromatographic separation system connected to an IDX High Resolution Mass Spectrometer (Thermo Scientific). The HILIC positive and negative data sets were processed via Compound Discoverer 3.2 according to the following settings: untargeted metabolomic workflow with online database: mass tolerance 10 ppm, maximum shift 0.3 min, alignment model adaptive curve, minimum intensity 500 K, S/N threshold 3, compound consolidation, mass tolerance 10 ppm, RT tolerance 0.3 min. Database matching was performed at MS2 level using Thermo scientific m/z cloud with a similar index of 80% or better.

### Statistical analysis

All statistical analyses and visualisations were performed in RStudio [28]. Data were assessed for normality by visualising Q–Q plots and performing Shapiro–Wilk tests before statistical analysis. If data were assessed to be non-normally distributed, then non-parametric statistical tests were performed.

Excluding the metagenomic and metabolomic data, changes from baseline within study phases and differences between study phases were assessed using mixed-effects models. In all models, age, BMI, habitual alcohol intake and diet order were included as fixed effects, with the participant as the random effect.

For gut microbial analysis, Bray–Curtis dissimilarity was performed to assess beta diversity and ordination plots generated for visualisation. Differences within and between study phases in beta diversity were compared using a permutational multivariate analysis of variance. Alpha diversity was measured using Shannon index, inverse Simpson index, Fisher’s index and species richness (CHAO1 estimates). As microbiome sequencing data can contain a considerable number of zeros, we applied geometric mean of pairwise ratio (GMPR), a normalization method for zero-inflated data [29]. Using the normalised data, abundances of taxonomic ranks (i.e. phyla, genus) were determined. Changes in alpha diversity metrics and bacterial taxonomic abundance within and differences between study phases were compared using generalised mixed-effects models with participants as a random-effects factor. The models were adjusted for age, BMI and habitual alcohol intake; factors which can impact the microbiome, as well as the interaction of the randomisation order to determine any carry over effects. *P* values were adjusted using the Benjamini and Hochberg false discovery rate [30].

For metabolomics, multivariate statistical analysis was performed using MetaboAnalyst 5.0 [31]. Partial least squares discriminant analysis (PLS-DA) was performed to determine changes in metabolite profiles within and differences between study phases. The variable importance in projection (VIP) > 1.5 was taken to identify the features significantly differentiating within and between study phases, then the fold change ratio was obtained for each feature. Hierarchical cluster analysis heat maps were obtained using ward clustering algorithm and Euclidean distance calculation to further confirm the results of PLS-DA and to show the distribution of metabolites among all individuals. Taking the VIPs > 1.5 between diets, pathway analyses were performed to reveal which pathways were enriched in the diets. Within the metabolomic datasets, potential genotoxins of interest were identified, including the bile acids apocholic and 7-ketodeoxycholic in stool, and p-cresol sulphate in urine. To assess the differences within and between study phases in the excretion of these genotoxins, mixed-effects models were implemented as outlined above.

For all statistical analyses, a *P* < 0.05 was defined significant.

## Results

### Participants

Participant enrolment began on 1 June 2019 and continued through to 1 December 2019. The date of final follow-up data collection was 29 January 2020. The Consort diagram in Supplementary Material: Fig. 1 depicts the flow of participants through the Mycomeat Study. Baseline characteristics of the participants who completed the study are shown in Table 1

**Table 1:** Participants’ baseline characteristics (*n* = 20)^a^

Age, y	30.4 ± 7.92
Anthropometric parameters	
Weight, kg	80.6 ± 10.90
BMI, kg/m^2^	24.0 ± 2.87
Waist circumference, cm	86.9 ± 8.16
Body fat, %	15.5 ± 5.56
Trunk fat, %	16.3 ± 6.90
Systolic blood pressure, mmHg	126 ± 12.2
Diastolic blood pressure, mmHg	71.5 ± 9.27
Blood parameters	
Total cholesterol, mmol/L	4.32 ± 0.83
HDL cholesterol, mmol/L	1.57 ± 0.43
LDL cholesterol, mmol/L	2.29 ± 0.88
Triglycerides, mmol/L	0.89 ± 0.46
Glucose, mmol/L	4.86 ± 0.45
Dietary intake	
Energy, kcal/d	2515.59 ± 754.85
Protein, g/d	126.44 ± 45.42
Protein, % of energy	20.10 ± 7.22
Carbohydrate, g/d	281.20 ± 81.41
Carbohydrate, % of energy	44.71 ± 12.94
Fat, g/d	98.37 ± 46.75
Fat, % of energy	35.19 ± 16.73
Saturated fat, g/d	35.94 ± 21.22
Saturated fat, % of energy	12.86 ± 7.59
Fibre, g/d	26.16 ± 7.79
Sodium, mg/d	2853.02 ± 1243.93
Total meat intake, g/d	221.60 ± 176.87
Red and processed meat intake g/d	80.25 ± 83.47
Mycoprotein intake, g/d	0 ± 0

^a^Data are presented as means ± SDs

### Diet

This intervention involved changes in the composition of the volunteers’ diets across both study arms. During the Meat phase, volunteers’ average daily meat consumption increased from baseline by 18 g, and red and processed meat intake by 160 g, whilst during the Mycoprotein phase, individuals did not consume any meat and increased their consumption of mycoprotein by an average 240 g per day from baseline. Mean self-reported intake of total energy, protein, fat, saturated fat and sodium were not significantly changed from baseline by either diet. The Mycoprotein phase was associated with higher self-reported fibre intake, both from baseline (+ 16.99 ± 2.79 g/day, *P* < 0.001) and compared to the Meat phase (+ 16.74 ± 3.65 g/day, *P* < 0.001) (Supplementary Material: Table 2).

### Faecal water genotoxicity

**Fig. 2:**
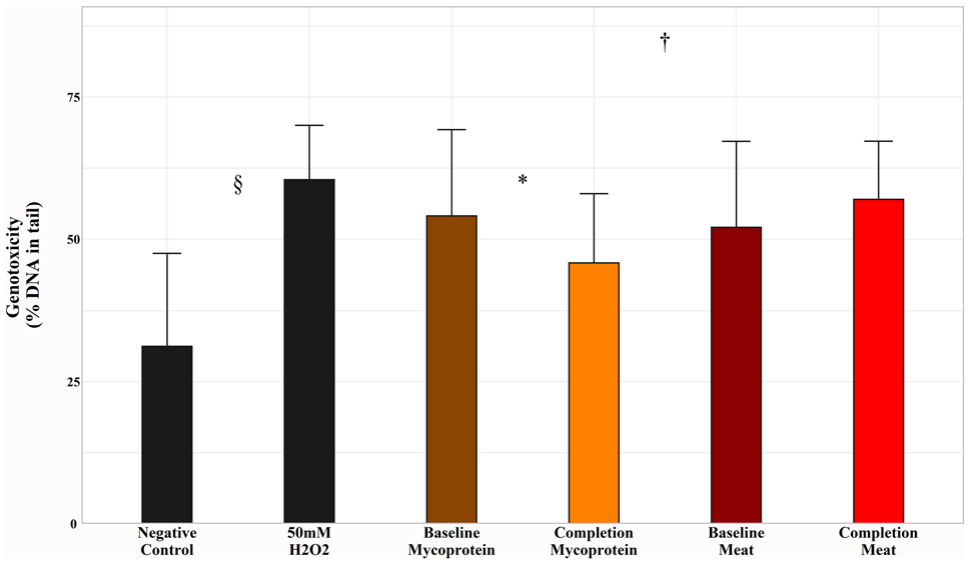
Effects of Meat and Mycoprotein phases on faecal genotoxicity, assessed by percentage (%) DNA in tail following exposure to faecal water. Data represent a mean of four wells per treatment. 50 uM H_2_O_2_ and a carrier control were included as inter-assay controls, and *t* tests revealed a significant difference between 50 uM H_2_O_2_ and carrier control (*P* = 0.02). Mycoprotein phase: change from baseline, − 8.28 ± 3.60%, *P* = 0.05. Meat phase: change from baseline, + 4.91 ± 2.65%, *P* = 0.09; Difference in study phase effects, 13.19 ± 4.41%, *P* = 0.01. Error bars represent standard deviation. Changes within study phases and differences between study phases assessed using mixed-effects models (*P* ˂0.05 considered significant). *Indicates significant difference from baseline within the Mycoprotein study phase. #Indicates significant difference from baseline within the Meat study phase. †Indicates significant difference between the Mycoprotein and Meat study phase effects. §Indicates significant difference between 50 uM H_2_O_2_ and carrier control

Faecal water genotoxicity significantly reduced from baseline after the Mycoprotein phase (− 8.28 ± 3.60% DNA in tail, *P* = 0.05), while following the Meat phase, there was a non-significant increase (+ 4.91 ± 2.65% DNA in tail, *P* = 0.09). The difference between these changes was also significant (13.19 ± 4.41% DNA in tail, *P* = 0.01) (Fig. 2).

### Potential genotoxins

**Fig. 3:**
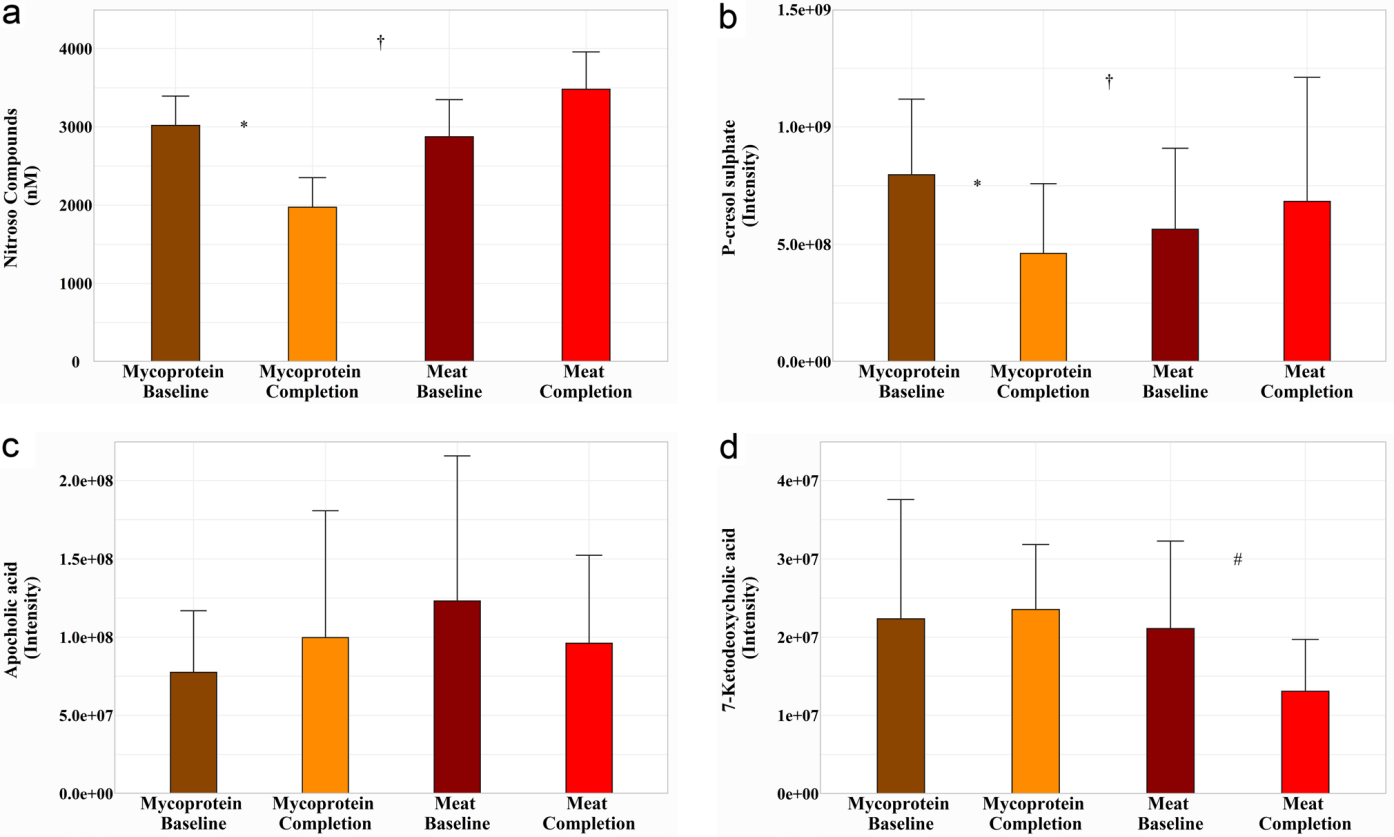
Effects of Meat and Mycoprotein phases on potential genotoxins. **a** Faecal nitroso compounds. Presented in nmol. Mycoprotein phase: change from baseline, − 1044.00 ± 377.00 nmol, *P* = 0.02. Meat phase: change from baseline, + 609.50 ± 541.00 nmol, *P* = 0.20; Difference in the study phase effects, 1653.50 ± 677.00 nmol, *P* = 0.01. **b** Urinary P-cresol sulphate. Presented in intensity. Mycoprotein phase: change from baseline, -3.35 × 10^8^ ± 9.8 × 10^8^intensity, *P* = 0.002; Meat phase: change from baseline, + 1.19 × 10^8^ ± 1.41 × 10^8^intensity, *P* = 0.40. Difference in study phase effects, 4.54 × 10^8^ ± 1.89 × 10^8^intensity, *P* = 0.02. **c** Faecal apocholic acid. Presented in intensity. Mycoprotein phase: change from baseline, + 2.23 × 10^7^ ± 2.01 × 10^7^intensity, *P* = 0.27. Meat phase: change from baseline, −2.70 × 10^7^ ± 2.42 × 10^7^intensity, *P* = 0.27. Difference in study phase effects, 4.93 × 10^7^ ± 3.37 × 10^7^intensity, *P* = 0.15. **d** Faecal 7-ketodeoxycholic acid. Presented in intensity. Mycoprotein phase: change from baseline, + 2.86 × 10^7^ ± 1.89 × 10^7^intensity, *P* = 0.82. Meat phase: change from baseline,  −8.03 × 10^6^ ± 2.91 × 10^6^intensity, *P* = 0.009. Difference in study phase effects, 3.66 × 10^7^ ± 2.00 × 10^7^intensity, *P* = 0.17. Error bars represent standard deviation. For all data, changes within study phases and differences between study phases assessed using mixed-effects models (*P* ˂0.05 considered significant). *Indicates significant difference from baseline within the Mycoprotein study phase. #Indicates significant difference from baseline within the Meat study phase. †Indicates significant difference between Mycoprotein and Meat study phase effects

Faecal NOC increased after the Meat phase (*P* = 0.20), whereas there was a significant reduction following the Mycoprotein phase (*P* = 0.007). The difference between the diet effects was also statistically significant (*P* = 0.01) (Fig. 3a). As well as NOC, other potential genotoxins were identified in the metabolomics data: p-cresol sulphate in urine and the bile acids apocholic and 7-ketodeoxycholic in stool.

Following the Mycoprotein phase, there was a significant reduction in urinary p-cresol sulphate excretion (*P* = 0.002), and with an increase after the Meat phase (*P* = 0.40), the difference between diet effects was also significant (*P* = 0.02) (Fig. 3b). Apocholic acid increased in stool from baseline after the Mycoprotein phase (*P* = 0.27) and reduced following the Meat phase (*P* = 0.27), with the difference between diet effects not significant (*P* = 0.15) (Fig. 3c). 7-Ketodeoxycholic acid followed a similar pattern, increasing after the Mycoprotein phase (*P* = 0.82) and reducing after the Meat phase (*P* = 0.009), the difference between diet effects was also not significant (*P* = 0.17) (Fig. 3d).

### Microbial composition

**Fig. 4:**
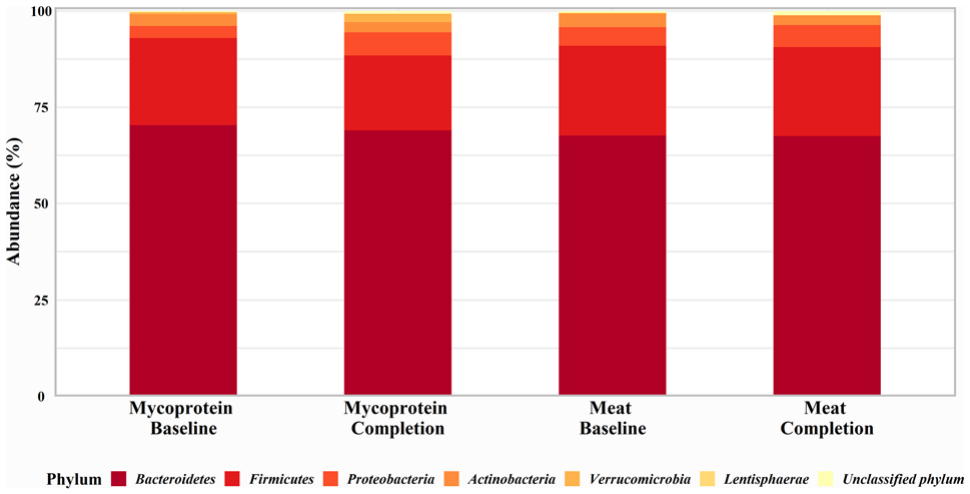
Effects of Meat and Mycoprotein phases on gut microbial phylum composition. Gut microbial phylum composition at baseline and completion for both Meat and Mycoprotein phases. There were significant increases in *Proteobacteria* (*P* = 0.004) and *Verrucomicrobia* (*P* = 0.02) following the Mycoprotein phase and a significant reduction in *Verrucomicrobia* (*P* = 0.04) after the Meat phase. The difference in diet effects on *Verrucomicrobia* (*P* = 0.03) was also significant. Changes within study phases and differences between study phases assessed using generalised mixed-effects models (*P* ˂0.05 considered significant)

Neither intervention affected alpha or beta diversity (data not shown). Bacterial phylotype analysis revealed significant changes in the relative abundances of bacterial communities. There were significant increases in the relative abundance of the bacterial phyla Proteobacteria (*P* = 0.004) and Verrucomicrobia (*P* = 0.02) following Mycoprotein, while Verrucomicrobia significantly reduced after Meat (*P* = 0.04), the difference between diet effects on Verrucomicrobia was also significant (*P* = 0.03) (Fig. 4).

**Fig. 5:**
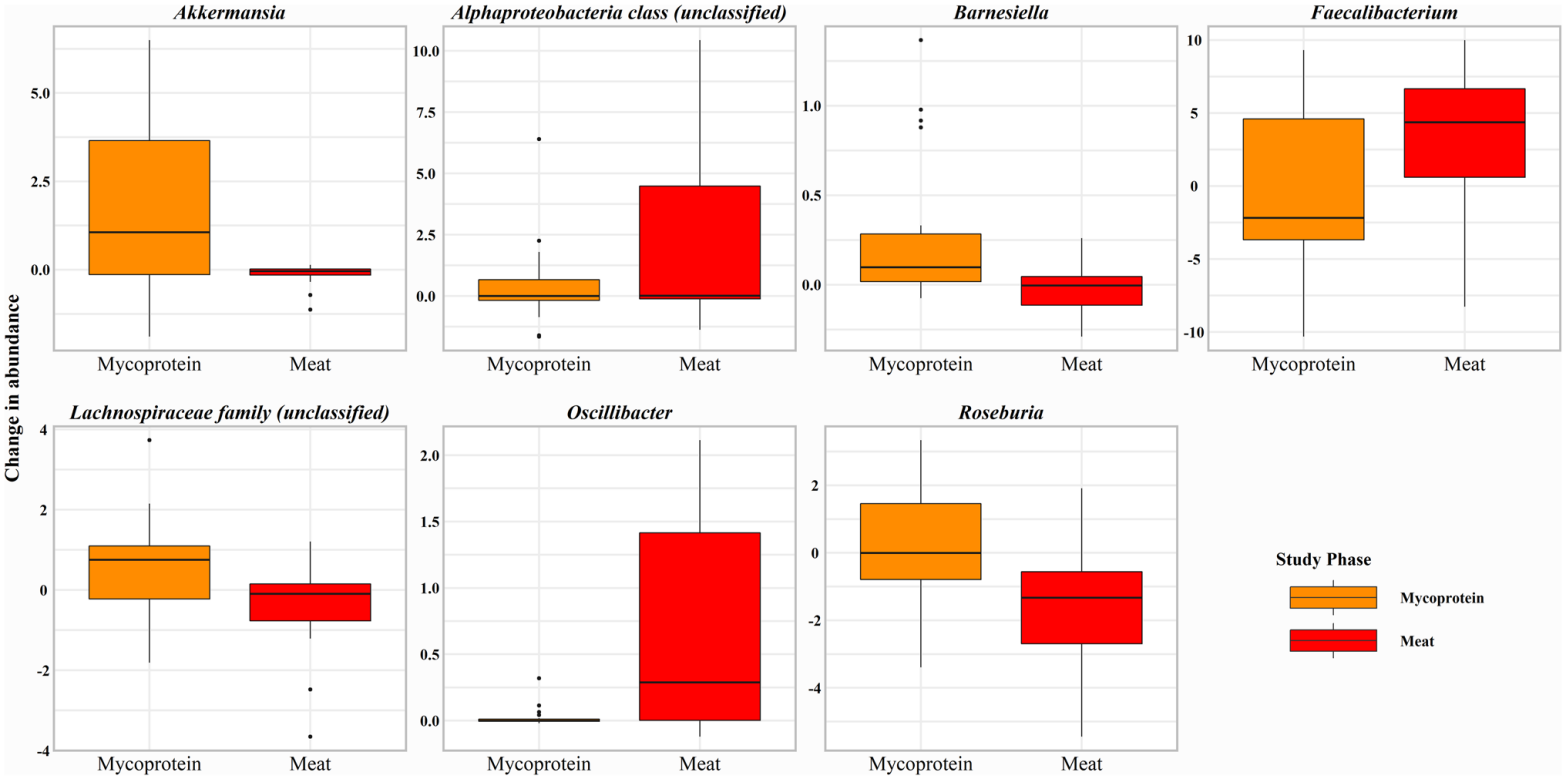
Effects of Meat and Mycoprotein phases on gut microbial genera. Gut microbial genera identified as significant for difference between study phase effects. Differences between study phases assessed using generalised mixed-effects models (*P* ˂0.05 considered significant)

**Fig. 6:**
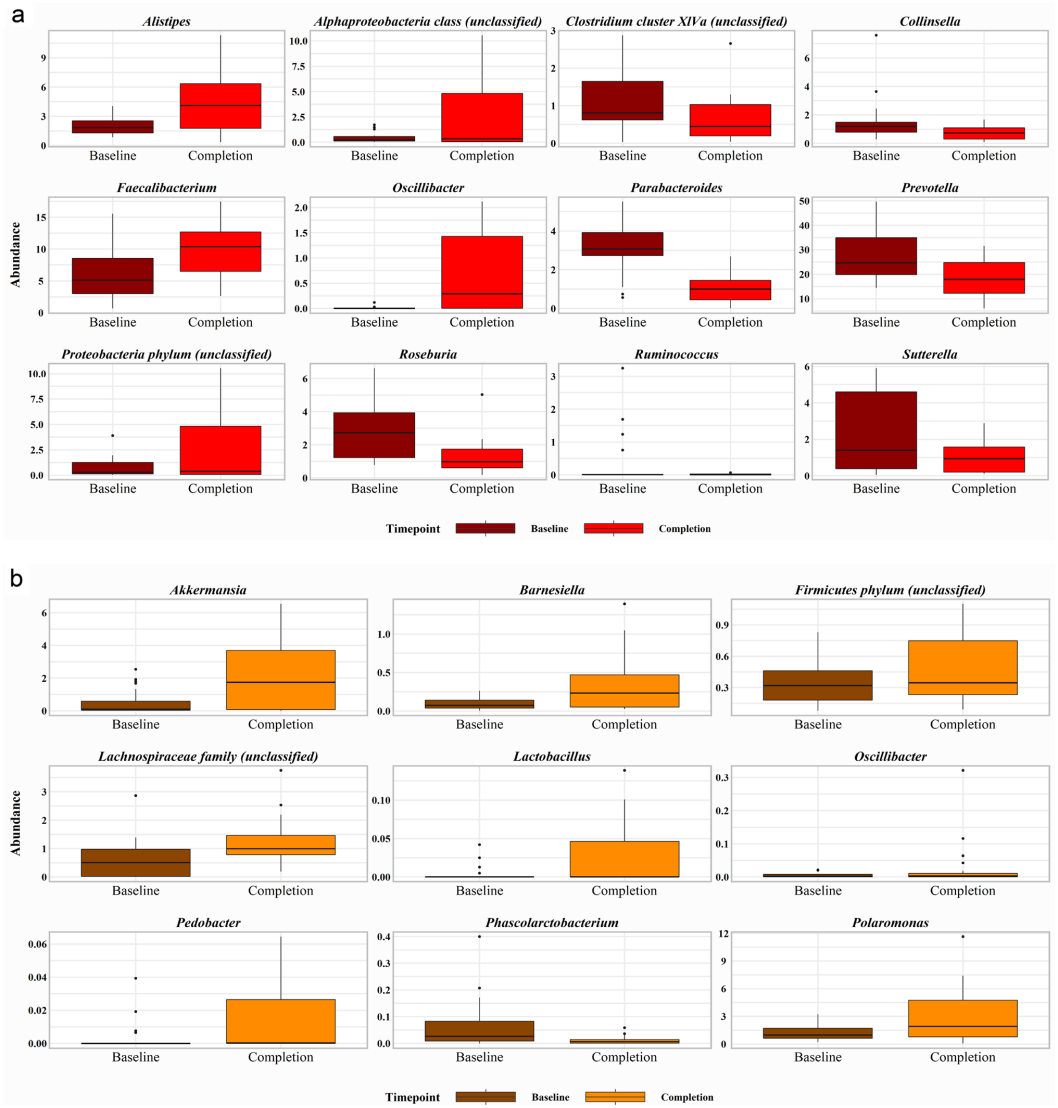
Effects of Meat and Mycoprotein phases on gut microbial genera. **a** Gut microbial genera identified as significant for change in abundance from baseline after the Mycoprotein phase. **b** Gut microbial genera identified as significant for change in abundance from baseline after the Meat phase. Changes within study phases assessed using generalised mixed-effects models (*P* ˂0.05 considered significant)

At the genera level, *Akkermansia* significantly increased following the Mycoprotein phase (*P* = 0.02), while there was a reduction in abundance after the Meat phase, the difference between diets being significant (*P* = 0.03). Mycoprotein and Meat phases had opposing effects on *Roseburia,* which increased after Mycoprotein (*P* < 0.001) and reduced following Meat (*P* < 0.001), with the difference between the diets being highly significant (*P* < 0.001). The diets also had contrasting influences on *Faecalibacterium*, which significantly increased after Meat (*P* = 0.006) and reduced following the Mycoprotein phase, with the difference between diets also being significant (*P* = 0.02). *Oscillibacter* was significantly increased following the Meat phase compared to the Mycoprotein phase (*P* = 0.004). Other notable effects were a significant reduction in *Ruminococcus* following the Meat phase (*P* = 0.03) and a significant increase in *Lactobacillus* after the Mycoprotein phase (*P* = 0.05). The genera significantly affected within the study phases are shown in Fig. 5 and those significantly different between study phases in Fig. 6.

In total, the analysis identified 392 OTUs in the samples. The OTUs identified as significantly different within and between study phases are included in the Supplementary Material: Table 3.

### Faecal volatile compounds

Total SCFA excretion increased following the Mycoprotein phase, with an increase in all quantified SCFA, none of which reached statistical significance (Table 2). In contrast, there was a non-significant reduction in total SCFA after the Meat phase, with reductions across all SCFA quantified, none which were significant. Valerate was the only SCFA to be significantly different between study phases (144.31 ± 53.76 μg/g, *P* = 0.02). Total BCFA excretion was reduced following both diets, with a significant reduction after the Meat phase (− 190.15 ± 87.24 μg/g, *P* = 0.01). The difference between diets also reached statistical significance for BCFA quantified, as well as total BCFA (151.81 ± 86.67 μg/g, *P* = 0.04). Both diets caused reductions in faecal cresol, with increases in phenol. The effects within and between diets did not reach significance.

**Table 2:** Effects of 2-wk dietary interventions on faecal volatile compounds (*n* = 20)^a,b^

Variable	Mean change from baseline, meat	*P*^c^	Mean change from baseline, mycoprotein	*P*^c^	Overall difference, meat and mycoprotein	*P*^c^
Short chain fatty acids						
Acetate	− 493.25 ± 233.32	0.76	+ 874.20 ± 671.69	0.26	+ 1367.45 ± 951.47	0.20
Propionate	− 313.35 ± 304.54	0.40	+ 637.10 ± 434.28	0.29	+ 950.45 ± 442.45	0.09
Butyrate	− 173.90 ± 170.30	0.66	+ 147.65 ± 117.99	0.74	+ 321.55 ± 316.92	0.54
Valerate	− 63.24 ± 50.68	0.12	+ 81.07 ± 58.72	0.39	+ 144.31 ± 53.76	0.02†
Hexanoate	− 13.05 ± 10.91	0.51	+ 20.16 ± 15.22	0.19	+ 33.21 ± 22.34	0.17
Total SCFA	− 1056.79 ± 806.37	0.58	+ 1760.17 ± 1229.62	0.34	+ 2816.96 ± 1749.88	0.17
Branch chain fatty acids						
Isobutyrate	− 98.10 ± 42.65	0.01*	− 13.29 ± 9.22	0.63	+ 84.81 ± 46.40	0.04†
Isovalerate	− 92.05 ± 45.06	0.02*	− 25.05 ± 23.46	0.42	+ 67.00 ± 41.25	0.05†
Total BCFA	− 190.15 ± 87.24	0.01*	− 38.34 ± 32.04	0.52	+ 151.81 ± 86.67	0.04†
Proteolytic end products						
Cresol	− 19.83 ± 17.25	0.18	− 12.79 ± 9.71	0.31	+ 7.04 ± 6.90	0.56
Phenol	+ 1.88 ± 1.16	0.15	+ 3.52 ± 1.72	0.40	− 1.64 ± 0.55	0.83

*SCFA* short chain fatty acids, *BCFA* branch chain fatty acids

*Mean change significantly different from baseline

†Mean change significantly different between mycoprotein and meat dietary periods

^a^Values are presented as least square means ± SEs. Differences between variables at the beginning and end of each diet are shown. The overall difference column shows the differences between variables at the end of the Mycoprotein phase compared with the end of the Meat phase. *P* values were calculated for changes within and differences between study phases using mixed-effects models

^b^Results are expressed in μg/g

^c^A *P* ˂0.05 was considered significant

### Metabolomics

A total of 1214 faecal metabolites were detected within the samples. PLS-DA between diets revealed a small distinction by the primary and secondary components (Supplementary Material: Fig. 2). Together, these two components accounted for ~ 26.6% of the variability (9.6 and 17.8% for the first and second components, respectively). Metabolites considered a VIP > 1.5 between diets are included in Supplementary Material: Table 4. Pathway analysis using the metabolites with VIP > 1.5 revealed thiamine metabolism was enriched, as well as pathways of fatty acid beta oxidation and carnitine synthesis following the Mycoprotein phase, whereas after the Meat phase, sphingolipid metabolism and purine metabolism were enriched (Supplementary Material: Fig. 2).

In urine, a total of 1,302 metabolites were detected within the samples. PLS-DA revealed a distinction between the diets by the primary and secondary components, however, with no defined clustering (Supplementary Material: Fig. 3). Together, these two components accounted for ~ 15.27% of the variability (7.73 and 7.54% for the first and second components, respectively). The metabolites considered a VIP > 1.5 are included in Supplementary Material: Table 5. Pathway analysis using the metabolites with VIP > 1.5 revealed thiamine metabolism was enriched after the Mycoprotein phase, followed by ammonia recycling, beta-alanine, -histidine, -glycine and -serine metabolism. However, taurine and hypotaurine metabolism was enriched the greatest after the Meat phase, followed by beta oxidation of very long chain fatty acids, phospholipid, steroid and bile acid biosynthesis. Both study phases led to an enrichment in the oxidation of BCFA (Supplementary Material: Fig. 3).

## Discussion

The present study investigated the impact of replacing a high red and processed meat diet with mycoprotein on faecal water genotoxicity and markers related to intestinal health. We recognise faecal water genotoxicity is poorly validated against tumour incidence in humans; however, it has been widely used as a non-invasive surrogate risk marker in short-term interventions exploring dietary components (notably meat) and CRC risk [5, 32, 33]. Further, it predicts subsequent tumour incidence in rodent models [34] and is used on the well-founded assumption that DNA damage is an initiating event in carcinogenesis [35], and therefore high genotoxic exposures may be viewed as a risk factor for neoplasia.

We observed that the Meat phase increased faecal water genotoxicity which corroborates previous findings [5, 21]. We also observed that the Mycoprotein phase significantly reduced genotoxicity, both relative to the Meat phase and to baseline (i.e., a standard western diet). This is a novel finding which suggests consuming mycoprotein may be protective against DNA damage, either via a displacement effect on harmful constituents of the diet, or independently via the introduction of antigenotoxic factors in the gut. Consistent with earlier studies, we also observed an increase in faecal NOC following the Meat phase, which, akin to faecal water genotoxicity, was significantly reduced by the Mycoprotein phase. The close association between genotoxicity and NOC suggests the latter as the predominant contributor to the former.

Both diets significantly influenced the composition of the gut microbiota. The most notable changes were increases in the relative abundance of *Lactobacilli*, *Roseburia,* and *Akkermansia* following the Mycoprotein phase. In models, *Lactobacilli* consistently exert significant protection against chemically induced tumours [36]. In vitro studies suggest one of the mechanisms for this anticancer effect may be via binding and chemical modification of NOC [37]. It is also evidenced that certain *Lactobacilli* augment intestinal barrier function by improving tight junction integrity [38] and enhancing colonic mucin production [39], conferring a further layer of defence against genotoxic insult. While this study, to our knowledge, is the first to report increases in *Lactobacilli* following mycoprotein consumption, it suggests that the fibre fraction of mycoprotein may have prebiotic potential [40]. The butyrate-producing *Roseburia* were also increased in relative abundance following the Mycoprotein phase, but decreased following the Meat phase. *Roseburia* have been associated with suppression of gut inflammatory processes [41] and are reduced in both inflammatory bowel disease [42] and CRC [43]. Butyrate is a potent anti-neoplastic agent in the gut [44]. *Akkermansia* is an abundant inhabitant of the human gut, degrading intestinal mucin which enhances cell turnover, in addition to priming trophic chains [45]. Further, propionic and acetic acids are metabolic by-products from the degradation of mucins, which can be absorbed and utilised by host tissue for energy or leveraged by other species to produce butyrate [45].

In contrast, the Meat phase caused the enrichment of the bile-resistant, putrefactive *Alistipes* and *Oscillibacter.* This might be an adaptive response to bile, or due to a greater proclivity for digestion-resistant myofibrillar protein arriving in the gut from meat versus mycoprotein. Acute feeding studies with mycoprotein lead to very high muscle protein synthesis rates, suggesting a high bioavailability of mycoprotein, which may culminate in low levels of dietary protein reaching the colon [46]. While observational data are conflicting, with studies showing *Alistipes* to be associated with both health and disease [47], *Oscillobacter* has been linked with weight gain, metabolic dysfunction and a leaky gut [48].

Prior to our study, gut fermentation of mycoprotein has only been reported in an in vitro model, which found an increase in total SCFA production [15]. We report increases in the faecal concentrations of all quantified SCFA following the Mycoprotein phase, which we attribute to the difference in fibre intake relative to participants’ habitual diet (+ 16.99 g day^−1^). The increased load of SCFA coincides with the enrichment of SCFA-producing genera following the Mycoprotein phase. Future work may consider a mycoprotein vs non-mycoprotein diet matched for fibre intake to elucidate if the 1,3/1,6 β-glucan/chitin present in mycoprotein is associated with a different SCFA response to other functional fibres. The reduction in SCFA excretion following the Meat phase was expected; diets rich in animal protein and lower in fibre are characterised by lower faecal SCFA compared to diets higher in fibre and lower in animal protein [49]. We also report reductions in BCFA following both diets. BCFA are considered markers of proteolytic fermentation [50] and concentrations tend to rise with increased protein intake [51]. Our observations could be due to the reduction in participants’ self-reported protein intake from baseline during intervention diets (− 11.41 g/day mycoprotein; − 8.61 g/day meat). There was also a significant difference in BCFA excretion between diets, with higher values following the Mycoprotein phase despite lower self-reported protein intake than the Meat phase. The difference in animal protein may not be a factor, as previous work found no differences in BCFA between vegans and omnivores [52]. The concentrations of excreted BCFA correlate weakly with the abundance of the largely saccharolytic genera *Blautia* (isobutyrate *r* = 0.235, *P* < 0.05, and isovalerate *r* = 0.229, *P* < 0.05), *Coprococcus* (isobutyrate *r* = 0.243, *P* < 0.05 and isovalerate *r* = 0.252, *P* < 0.05) and *Odoribacter* (isovalerate *r* = 0.225 *P* < 0.05). The higher concentrations of BCFA with the Mycoprotein relative to the Meat phase may therefore simply reflect higher saccharolytic microbial activity on that arm in response to the relative availability of fibre.

Applying untargeted metabolomics, we were able to decipher the predominant metabolic pathways in urine and stool. We found an enrichment in sphingolipid metabolism after the Meat phase. Sphingolipids are important mammalian signalling molecules with various biological activities, and importantly increased sphingolipid metabolism may be a response to DNA damage [53].

The rich metabolomic datasets also enabled the identification of other potential genotoxins as well as our targeted NOC analysis. We identified p-cresol sulphate in urine, a product of tyrosine fermentation in the gut [54], which is absorbed and sulphated for excretion in urine [55]. Studies have demonstrated p-cresol reaches genotoxic concentrations in in vitro gut fermentation models, and notably can predict the genotoxicity of the fermentation supernatant [56]. Like faecal genotoxicity and NOC, urinary p-cresol sulphate was increased by the Meat phase and significantly reduced by the Mycoprotein phase. These findings are further indicative of negative and positive effects of meat and mycoprotein, respectively. We also identified the bile acids apocholic and 7-ketodeoxycholic in stool. 7α-dehydroxylating bacteria convert and activate bile acids in the gut [57]. The transformed secondary bile acids have the potential to induce oxidative stress and DNA damage [58, 59] and are implicated in tumorigenesis [60]. We observed reductions in these bile acids after the Meat and increases following the Mycoprotein phase. Thus, we would argue they are not the main contributors to the genotoxic load in the faecal samples. Bile acid metabolism plays a predominant role in cholesterol homeostasis [61], and increased bile acids in stool may be indicative of mycoprotein’s ability to bind and sequester bile acids [62]. This may be a mechanism underpinning observations of cholesterol reduction by mycoprotein [63–65].

The present study has several strengths. We used a randomised crossover control investigator-blind study design, thus significantly reducing bias. The meat and mycoprotein products included are regularly consumed by the public, and the dose is achievable in a real-world setting. We also incorporated a variety of products in the dietary regimens to aid participant compliance, and to reflect the variety in real-world food exposures. It is noteworthy that once randomised to the study, we encountered no participant dropouts, which may be in part due to this approach. The Thermo IDX LCMS platform uses automated data acquisition and peak annotation resulting in improved metabolite profiling efficacy in comparison to alternative platforms. This allowed the ability to obtain a greater number of identifiable metabolites, which in turn enabled a rich dataset to determine metabolic pathways hitherto relatively unexplored in this type of study.

Our work also has limitations. The cohort included exclusively healthy, non-obese adult males. This cohort was selected to control external factors, but future studies might use larger, more diverse cohorts. We advised participants to avoid certain foods and supplements; however, we did not control dietary intake other than the supplied study products. Other dietary factors during the study phases may have influenced our findings. 16S amplicon sequencing was used for microbial analysis which limits the coverage to genus. We also collected single-day faecal samples, but the stool microbiome can show intraday variation.

In conclusion, this work demonstrates that substituting red and processed meat for the meat alternative mycoprotein reduces faecal genotoxins and the genotoxic load and increases SCFA production, as well as the abundance of beneficial genera in the human gut. Thus, mycoprotein may be considered a good alternative to meat when consumed as part of a balanced diet in the context of gut health and long-term CRC risk.

## Supplementary Information

Below is the link to the electronic supplementary material.

Supplementary file1 (DOCX 716 KB)

## Data Availability

Data described in the manuscript will be made publicly and freely available without restriction at https://doi.org/10.25398/rd.northumbria.c.6010252.

## References

[1] Bernstein AM, Song M, Zhang X, Pan A, Wang M, Fuchs CS (2015). Processed and unprocessed red meat and risk of colorectal cancer: analysis by tumor location and modification by time. PLoS ONE.

[2] Carr PR, Holleczek B, Stegmaier C, Brenner H, Hoffmeister M (2017). Meat intake and risk of colorectal polyps: results from a large population-based screening study in Germany. Am J Clin Nutr.

[3] Cross AJ, Leitzmann MF, Gail MH, Hollenbeck AR, Schatzkin A, Sinha R (2007). A prospective study of red and processed meat intake in relation to cancer risk. PLoS Med.

[4] Key TJ, Bradbury KE, Murphy N (2019). Diet and colorectal cancer in UK Biobank: a prospective study. Int J Epidemiol.

[5] Hughes R, Pollock JR, Bingham S (2002). Effect of vegetables, tea, and soy on endogenous N-nitrosation, fecal ammonia, and fecal water genotoxicity during a high red meat diet in humans. Nutr Cancer.

[6] Joosen AM, Lecommandeur E, Kuhnle GG, Aspinall SM, Kap L, Rodwell SA (2010). Effect of dietary meat and fish on endogenous nitrosation, inflammation and genotoxicity of faecal water. Mutagenesis.

[7] Gao J, Guo X, Wei W, Li R, Hu K, Liu X (2021). The association of fried meat consumption with the gut microbiota and fecal metabolites and its impact on glucose homoeostasis, intestinal endotoxin levels, and systemic inflammation: a randomized controlled-feeding trial. Diabetes Care.

[8] Bouvard V, Loomis D, Guyton KZ, Grosse Y, Ghissassi FE, Benbrahim-Tallaa L (2015). Carcinogenicity of consumption of red and processed meat. Lancet Oncol.

[9] Willett W, Rockström J, Loken B, Springmann M, Lang T, Vermeulen S (2019). Food in the anthropocene: the EAT-lancet commission on healthy diets from sustainable food systems. Lancet.

[10] Sanchez-Sabate R, Sabaté J (2019). Consumer attitudes towards environmental concerns of meat consumption: a systematic review. Int J Environ Res Public Health.

[11] Kumar P, Chatli MK, Mehta N, Singh P, Malav OP, Verma AK (2017). Meat analogues: Health promising sustainable meat substitutes. Crit Rev Food Sci Nutr.

[12] Hu FB, Otis BO, McCarthy G (2019). Can plant-based meat alternatives be part of a healthy and sustainable diet?. JAMA.

[13] Finnigan T, Needham L, Abbott C, editors (2017) Mycoprotein: a healthy new protein with a low environmental impact

[14] Derbyshire E, Ayoob K-T (2019). Mycoprotein: nutritional and Health properties. Nutr Today.

[15] Harris HC, Edwards CA, Morrison DJ (2019). Short chain fatty acid production from mycoprotein and mycoprotein fibre in an in vitro fermentation model. Nutrients.

[16] Wang X, Gibson GR, Costabile A, Sailer M, Theis S, Rastall RA (2019). Prebiotic supplementation of in vitro fecal fermentations inhibits proteolysis by gut bacteria, and host diet shapes gut bacterial metabolism and response to intervention. Appl Environ Microbiol.

[17] Cherta-Murillo A, Lett AM, Frampton J, Chambers ES, Finnigan TJA, Frost GS (2020). Effects of mycoprotein on glycaemic control and energy intake in humans: a systematic review. Br J Nutr.

[18] Coelho MOC, Monteyne AJ, Dunlop MV, Harris HC, Morrison DJ, Stephens FB (2020). Mycoprotein as a possible alternative source of dietary protein to support muscle and metabolic health. Nutr Rev.

[19] Hughes R, Cross AJ, Pollock JRA, Bingham S (2001). Dose-dependent effect of dietary meat on endogenous colonic N-nitrosation. Carcinogenesis.

[20] Collins SM, Gibson GR, Kennedy OB, Walton G, Rowland I, Commane DM (2021). Development of a prebiotic blend to influence in vitro fermentation effects, with a focus on propionate, in the gut. FEMS Microbiol Ecol.

[21] Hebels DG, Sveje KM, de Kok MC, van Herwijnen MH, Kuhnle GG, Engels LG (2012). Red meat intake-induced increases in fecal water genotoxicity correlate with pro-carcinogenic gene expression changes in the human colon. Food Chem Toxicol.

[22] Nutritics. *Education Edition, v566, Dublin, Nutritics, 2021.

[23] Ge J, Prasongtanakij S, Wood DK, Weingeist DM, Fessler J, Navasummrit P (2014). CometChip: a high-throughput 96-well platform for measuring DNA damage in microarrayed human cells. J Visualized Exp: JoVE.

[24] Kuhnle GGC, Story GW, Reda T, Mani AR, Moore KP, Lunn JC (2007). Diet-induced endogenous formation of nitroso compounds in the GI tract. Free Radic Biol Med.

[25] Kozich JJ, Westcott SL, Baxter NT, Highlander SK, Schloss PD (2013). Development of a dual-index sequencing strategy and curation pipeline for analyzing amplicon sequence data on the MiSeq Illumina sequencing platform. Appl Environ Microbiol.

[26] Caporaso JG, Lauber CL, Walters WA, Berg-Lyons D, Lozupone CA, Turnbaugh PJ (2011). Global patterns of 16S rRNA diversity at a depth of millions of sequences per sample. Proceed National Acad Sci United States of America.

[27] Edmands WMB, Ferrari P, Scalbert A (2014). Normalization to specific gravity prior to analysis improves information recovery from high resolution mass spectrometry metabolomic profiles of human urine. Anal Chem.

[28] R Core Team (2020) R: A language and environment for statistical computing: R Foundation for Statistical Computing

[29] Chen L, Reeve J, Zhang L, Huang S, Wang X, Chen J (2018). GMPR: a robust normalization method for zero-inflated count data with application to microbiome sequencing data. PeerJ.

[30] Benjamini Y, Hochberg Y (1995). Controlling the false discovery rate: a practical and powerful approach to multiple testing. J Royal Statistical Soc: Series B (Methodological).

[31] Pang Z, Chong J, Zhou G, de Lima Morais DA, Chang L, Barrette M (2021). MetaboAnalyst 5.0: narrowing the gap between raw spectra and functional insights. Nucleic Acids Res.

[32] Costabile A, Fava F, Röytiö H, Forssten SD, Olli K, Klievink J (2012). Impact of polydextrose on the faecal microbiota: a double-blind, crossover, placebo-controlled feeding study in healthy human subjects. Br J Nutr.

[33] Eid N, Osmanova H, Natchez C, Walton G, Costabile A, Gibson G (2015). Impact of palm date consumption on microbiota growth and large intestinal health: a randomised, controlled, cross-over, human intervention study. Br J Nutr.

[34] Klinder A, Forster A, Caderni G, Femia AP, Pool-Zobel BL (2004). Fecal water genotoxicity is predictive of tumor-preventive activities by inulin-like oligofructoses, Probiotics (*Lactobacillus rhamnosus* and *Bifidobacterium lactis*), and their synbiotic combination. Nutr Cancer.

[35] Alhmoud JF, Woolley JF, Al Moustafa A-E, Malki MI (2020). DNA damage/repair management in cancers. Cancers.

[36] Brasiel PGA, Dutra Luquetti SCP, Peluzio M, Novaes RD, Gonçalves RV (2020). Preclinical evidence of probiotics in colorectal carcinogenesis: a systematic review. Dig Dis Sci.

[37] Caldini G, Trotta F, Villarini M, Moretti M, Pasquini R, Scassellati-Sforzolini G (2005). Screening of potential lactobacilli antigenotoxicity by microbial and mammalian cell-based tests. Int J Food Microbiol.

[38] Karczewski J, Troost FJ, Konings I, Dekker J, Kleerebezem M, Brummer R-JM (2010). Regulation of human epithelial tight junction proteins by *Lactobacillus plantarum* in vivo and protective effects on the epithelial barrier. A J Physiol- Gastrointestinal Liver Physiol.

[39] Chen CM, Wu CC, Huang CL, Chang MY, Cheng SH, Lin CT (2021). *Lactobacillus plantarum* PS128 promotes intestinal motility, mucin production, and serotonin signaling in mice. Probiotics Antimicrob Proteins.

[40] Costabile A, Kolida S, Klinder A, Gietl E, Bäuerlein M, Frohberg C (2010). A double-blind, placebo-controlled, cross-over study to establish the bifidogenic effect of a very-long-chain inulin extracted from globe artichoke (*Cynara scolymus*) in healthy human subjects. Br J Nutr.

[41] Atarashi K, Tanoue T, Shima T, Imaoka A, Kuwahara T, Momose Y (2011). Induction of colonic regulatory T cells by indigenous clostridium species. Science.

[42] Takahashi K, Nishida A, Fujimoto T, Fujii M, Shioya M, Imaeda H (2016). Reduced abundance of butyrate-producing bacteria species in the fecal microbial community in Crohn’s disease. Digestion.

[43] Geng J, Fan H, Tang X, Zhai H, Zhang Z (2013). Diversified pattern of the human colorectal cancer microbiome. Gut Pathogens.

[44] Bernardazzi C, Xu H, Tong H, Laubitz D, Figliuolo da Paz V, Curiel L (2020). An indisputable role of NHE8 in mucosal protection. Am J Physiol Gastrointest Liver Physiol.

[45] de Vos WM (2017). Microbe Profile: Akkermansia muciniphila: a conserved intestinal symbiont that acts as the gatekeeper of our mucosa. Microbiology.

[46] Monteyne AJ, Coelho MOC, Porter C, Abdelrahman DR, Jameson TSO, Jackman SR (2020). Mycoprotein ingestion stimulates protein synthesis rates to a greater extent than milk protein in rested and exercised skeletal muscle of healthy young men: a randomized controlled trial. Am J Clin Nutr.

[47] Parker BJ, Wearsch PA, Veloo ACM, Rodriguez-Palacios A (2020). The genus alistipes: gut bacteria with emerging implications to inflammation, cancer, and mental health. Front Immunol.

[48] Lam YY, Ha CWY, Campbell CR, Mitchell AJ, Dinudom A, Oscarsson J (2012). Increased gut permeability and microbiota change associate with mesenteric fat inflammation and metabolic dysfunction in diet-induced obese mice. PLoS ONE.

[49] Ríos-Covián D, Ruas-Madiedo P, Margolles A, Gueimonde M, de Los Reyes-Gavilán CG, Salazar N (2016). Intestinal short chain fatty acids and their link with diet and human health. Front Microbiol.

[50] Windey K, De Preter V, Verbeke K (2012). Relevance of protein fermentation to gut health. Mol Nutr Food Res.

[51] Russell WR, Gratz SW, Duncan SH, Holtrop G, Ince J, Scobbie L (2011). High-protein, reduced-carbohydrate weight-loss diets promote metabolite profiles likely to be detrimental to colonic health. Am J Clin Nutr.

[52] Trefflich I, Dietrich S, Braune A, Abraham K, Weikert C (2021). Short- and branched-chain fatty acids as fecal markers for microbiota activity in vegans and omnivores. Nutrients.

[53] Xu R, Garcia-Barros M, Wen S, Li F, Lin C-L, Hannun YA (2017). Tumor suppressor p53 links ceramide metabolism to DNA damage response through alkaline ceramidase 2. Cell Death Differ.

[54] Saito Y, Sato T, Nomoto K, Tsuji H (2018). Identification of phenol- and p-cresol-producing intestinal bacteria by using media supplemented with tyrosine and its metabolites. FEMS Microbiol Ecol.

[55] Wikoff WR, Anfora AT, Liu J, Schultz PG, Lesley SA, Peters EC (2009). Metabolomics analysis reveals large effects of gut microflora on mammalian blood metabolites. Proc Natl Acad Sci.

[56] Al Hinai EA, Kullamethee P, Rowland IR, Swann J, Walton GE, Commane DM (2018). Modelling the role of microbial p-cresol in colorectal genotoxicity. Gut Microbes.

[57] Ridlon JM, Kang DJ, Hylemon PB (2006). Bile salt biotransformations by human intestinal bacteria. J Lipid Res.

[58] Dvorak K, Payne CM, Chavarria M, Ramsey L, Dvorakova B, Bernstein H (2007). Bile acids in combination with low pH induce oxidative stress and oxidative DNA damage: relevance to the pathogenesis of Barrett’s oesophagus. Gut.

[59] Lechner S, Müller-Ladner U, Schlottmann K, Jung B, McClelland M, Rüschoff J (2002). Bile acids mimic oxidative stress induced upregulation of thioredoxin reductase in colon cancer cell lines. Carcinogenesis.

[60] Ocvirk S, O'Keefe SJ (2017). Influence of bile acids on colorectal cancer risk: potential mechanisms mediated by Diet–Gut microbiota interactions. Curr Nutr Rep.

[61] Chiang JYL (2013). Bile acid metabolism and signaling. Compr Physiol.

[62] Colosimo R, Mulet-Cabero A-I, Warren FJ, Edwards CH, Finnigan TJA, Wilde PJ (2020). Mycoprotein ingredient structure reduces lipolysis and binds bile salts during simulated gastrointestinal digestion. Food Funct.

[63] Coelho MOC, Monteyne AJ, Dirks ML, Finnigan TJA, Stephens FB, Wall BT (2021). Daily mycoprotein consumption for 1 week does not affect insulin sensitivity or glycaemic control but modulates the plasma lipidome in healthy adults: a randomised controlled trial. Br J Nutr.

[64] Turnbull WH, Leeds AR, Edwards DG (1992). Mycoprotein reduces blood lipids in free-living subjects. Am J Clin Nutr.

[65] Turnbull WH, Leeds AR, Edwards GD (1990). Effect of mycoprotein on blood lipids. Am J Clin Nutr.

