# Supplementary material for: Substituting meat for mycoprotein reduces genotoxicity and increases the abundance of beneficial microbes in the gut: Mycomeat, a randomised crossover control trial
Source: Eur J Nutr. 2023 Jan 18;62(3):1479–92. doi: 10.1007/s00394-023-03088-x (PMC10030420; doi:10.1007/s00394-023-03088-x)
Supplement: Supplementary file 1 — Supplementary file1 (DOCX 716 KB) [file 394_2023_3088_MOESM1_ESM.docx]

**SUPPLEMENTARY MATERIAL**

**Substituting meat for mycoprotein reduces genotoxicity and increases the abundance of beneficial microbes in the gut: Mycomeat, a randomised crossover control trial**

**Figure 1.** Mycomeat CONSORT diagram

**Table 1i.** Nutrient profiles for Mycoprotein and Meat products

**Table 1ii.** Difference in nutritional composition between Mycoprotein and Meat products

**Table 2.** Difference in nutritional intake between diets, including provided study food (n = 20)

**Table 3.** Operational Taxonomic Units (OTUs) significantly affected by Meat and Mycoprotein (n = 20)

**Figure 2.** Comparison of faecal metabolome between Meat and Mycoprotein

**Table 4.** Faecal metabolites considered a VIP >1.5 between Meat and Mycoprotein.

**Figure 3.** Comparison of urine metabolome between Meat and Mycoprotein

**Table 5.** Urine metabolites considered a VIP >1.5 between Meat and Mycoprotein

**Figure 1. Mycomeat CONSORT**

3 Excluded

All 3 declined to participate

23 Individuals assessed for eligibility (n= 23)

20 Contributed to analyses

20 finished Phase 2
(Meat or Mycoprotein)

20 Randomly allocated to receive Meat or Mycoprotein in Phase 1

20 Randomised

20 finished Phase 1
(Meat or Mycoprotein)

20 Crossed over to receive Meat or Mycoprotein in Phase 2

| **Table 1i. Nutrient profiles for Mycoprotein and Meat products^1^** | | | | | | | | |
| --- | --- | --- | --- | --- | --- | --- | --- | --- |
| **Product^2,3^** | | **Calories, kcals** | **Carbohydrates, g** | **Protein, g** | **Total fat, g** | **Saturates, g** | **Fibre, g** | **Sodium, mg** |
| Mycoprotein products | |  |  |  |  |  |  |  |
| Peppered Steak | 269 | 12.0 | 28.1 | 11.8 | 5.3 | 13.0 | 1248 |  |
| Sausage | 475 | 25.4 | 26.9 | 26.6 | 3.4 | 13.2 | 1152 |  |
| Meat Free Ham Deli Slices | 295 | 12.5 | 39.8 | 6.5 | 2.9 | 13.0 | 864 |  |
| Gammon Steaks | 298 | 4.1 | 36.2 | 12.5 | 6.2 | 12.7 | 1440 |  |
| Bacon Style Slices | 514 | 13.0 | 24.2 | 37.7 | 2.6 | 13.4 | 1536 |  |
| Mince | 252 | 10.8 | 34.8 | 4.8 | 1.2 | 13.2 | 288 |  |
| Hot Dogs | 410 | 8.6 | 35.5 | 23.0 | 1.7 | 13.0 | 1248 |  |
| Meat products | |  |  |  |  |  |  |  |
| Beef Steak | 367 | 0 | 49.0 | 18.6 | 8.4 | 0 | 188 |  |
| Pork Sausage | 390 | 13.2 | 28.8 | 23.0 | 8.3 | 4.2 | 768 |  |
| Cold Cut Ham | 257 | 1.2 | 40.8 | 9.6 | 3.8 | 0 | 1536 |  |
| Gammon Steak | 360 | 0.9 | 52.2 | 16.4 | 6.7 | 0 | 1800 |  |
| Bacon Rashers | 272 | 0 | 30.7 | 16.3 | 6.6 | 0 | 1536 |  |
| Beef Mince | 200 | 0 | 31.9 | 6.7 | 3.4 | 0 | 134 |  |
| Hot Dogs | 540 | 1.2 | 31.2 | 45.6 | 19.7 | 0 | 1056 |  |
| ^1^Nutritent profiles obtained from Quorn Foods for Mycoprotein products and Asda supermarket for Meat products.  ^2^ Products included in 7 day rotation.Each product repeated twice over the 14 day diet phases. ^3^All products were supplied as 240g uncooked servings. | | | | | | | | |

| **Table 1ii. Difference in nutritional composition between Mycoprotein and Meat products^1^.** | | | | | |
| --- | --- | --- | --- | --- | --- |
|  | | Meat products | Mycoprotein products | Difference | *P***^2^** |
| **Energy** | |  |  |  |  |
| Content in daily portion, *kcal* | 341.00 ± 107.00 | 359.00 ± 102.00 | +18.00 ± 123 | 0.65 |  |
| **Protein** | |  |  |  |  |
| Content in daily portion, *g* | 37.80 ± 9.20 | 32.20 ± 5.56 | -5.60 ± 9.17 | 0.07 |  |
| Contribution to energy total, *%* | 48.10 ± 15.60 | 39.40 ± 14.00 | -8.70 ± 10.60 | 0.13 |  |
| **Carbohydrates** | |  |  |  |  |
| Content in daily portion, *g* | 2.36 ± 4.64 | 12.30 ± 6.28 | +9.94 ± 3.35 | ˂ 0.001 |  |
| Contribution to energy total, *%* | 2.48 ± 4.76 | 13.90 ± 5.64 | +11.42 ± 5.04 | ˂ 0.001 |  |
| **Fat** | |  |  |  |  |
| Content in daily portion, *g* | 19.50 ± 12.30 | 17.60 ± 11.50 | -1.90 ± 12.60 | 0.68 |  |
| Contribution to energy total, *%* | 47.70 ± 14.80 | 40.10 ± 16.80 | -7.60 ± 11.2 | 0.22 |  |
| **Saturated** **Fat** | |  |  |  |  |
| Content in daily portion, *g* | 8.11 ± 5.25 | 3.33 ± 1.76 | -4.78 ± 5.80 | 0.01 |  |
| **Fibre** | |  |  |  |  |
| Content in daily portion, *g* | 0.60 ± 1.53 | 13.10 ± 0.22 | +12.50 ± 1.49 | ˂ 0.001 |  |
| **Sodium** | |  |  |  |  |
| Content in daily portion, *mg* | 987.06 ± 631.66 | 1093.36 ± 398.67 | +106.03 ± 522.33 | 0.60 |  |
| **^1^** Daily averages for the intervention periods. Data is expressed as means ± SD. Differences between the supplied intervention food was tested with independent t-tests. The difference column shows the difference between the Mycoprotein products compared to the Meat products. **^2^** *P* > 0.05 is considered NS. | | | | |  |

| **Table 2. Difference in nutritional intake between Meat and Mycoprotein, including provided study food (*n* = 20)^1^** | | | | |
| --- | --- | --- | --- | --- |
| Nutrient | Meat | Mycoprotein | Difference^2^ | *P*^3^ |
| Energy, *kcal/d* | 2355.18 ± 553.32 | 2567.44 ± 796.97 | +212.26 ± 242.63 | 0.47 |
| Protein, *g/d* | 117.83 ± 31.26 | 115.03 ± 35.86 | -2.80 ± 11.92 | 0.78 |
| Carbohydrate, *g/d* | 243.82 ± 48.81 | 298.20 ± 104.37 | +54.38 ± 28.86 | 0.06 |
| Fat, *g/d* | 100.43 ± 39.95 | 98.98 ± 37.04 | -1.45 ± 13.51 | 0.68 |
| Saturated fat, *g/d* | 36.96 ± 19.32 | 36.92 ± 14.64 | -0.04 ± 6.01 | 0.79 |
| Fibre, *g/d* | 26.52 ± 8.40 | 43.26 ± 8.69 | +16.74 ± 2.92 | < 0.001 |
| Sodium, *mg/d* | 2714.25 ± 979.59 | 3229.12 ± 1405.28 | +514.87 ± 424.95 | 0.32 |
| ^1^Values are presented as means ± SDs. Nutritional intake calculated from 1-day food records using Nutritics nutritional analysis software. ^2^ Differences between nutrient intake during Mycoprotein compared to Meat presented as least square means ± SEs.  ^3^*P* values were calculated for differences between study phases using mixed effects models. A *P* ˂0.05 was considered significant. | | | | |

| **Table 3.** **Operational Taxonomic Units (OTUs) significantly affected by Meat and Mycoprotein (n = 20)** Table includes OTU number as well as microbial genera assigned to OTU. For OTUs where genera not assigned, the closest available known taxa is assigned (i.e. family). | | | |
| --- | --- | --- | --- |
| **Operational Taxonomic Units (OTU’s) significantly different following Mycoprotein** | | | |
| No. Operational Taxonomic Unit (OTU) | Bacterial Taxonomic Classification | Change in relative abundance from baseline*^1^* | *P^3^* |
| 1 | *Bacteroides* | +1.58 | 0.04 |
| 13 | *Polaromonas* | +1.80 | 0.03 |
| 15 | *Parabacteroides* | -1.54 | 0.01 |
| 19 | *Prevotella* | -1.48 | 0.006 |
| 25 | *Alistipes* | +1.11 | 0.001 |
| 27 | *Lachnospiraceae family (unclassified)* | +0.60 | 0.009 |
| 31 | *Oscillibacter* | +0.04 | 0.05 |
| 33 | *Akkermansia* | -1.66 | 0.007 |
| 34 | *Barnesiella* | +0.12 | 0.008 |
| 42 | *Phascolarctobacterium* | -0.07 | 0.01 |
| 44 | *Parabacteroides* | +0.56 | 0.002 |
| 50 | *Bacteroides* | +0.67 | 0.002 |
| 57 | *Ruminococcaceae family (unclassified)* | -0.046 | 0.03 |
| 91 | *Clostridium Cluster IV (unclassified)* | +0.001 | 0.01 |
| 95 | *Lactobacillus* | +0.02 | 0.05 |
| 102 | *Pedobacter* | +0.01 | 0.02 |
| 107 | *Prevotella* | -0.02 | 0.01 |
| 110 | *Bacteroidetes phylum (unclassified)* | -0.01 | 0.007 |
| 163 | *Clostridiales family (unclassified)* | -0.002 | 0.003 |
|  |  |  |  |
|  |  |  |  |
| **Table 3. (continued)**  **Operational Taxonomic Units (OTU’s) significantly different following Meat** | | | |
| No. Operational Taxonomic Unit (OTU) | Bacterial Taxonomic Classification | Change in relative abundance from baseline | *P^3^* |
| 2 | *Faecalibacterium* | +3.77 | 0.02 |
| 5 | *Bacteroides* | -2.14 | 0.04 |
| 9 | *Alistipes* | +1.94 | 0.01 |
| 10 | *Roseburia* | -2.01 | < 0.01 |
| 12 | *Bacteroides* | +0.85 | 0.03 |
| 14 | *Sutterella* | -1.29 | 0.02 |
| 15 | *Parabacteroides* | -2.01 | < 0.01 |
| 21 | *Bacteroides* | +1.71 | 0.03 |
| 27 | *Lachnospiraceae family (unclassified)* | -0.65 | 0.03 |
| 31 | *Oscillibacter* | +0.70 | 0.001 |
| 37 | *Prevotellaceae family (unclassified)* | +0.06 | 0.02 |
| 40 | *Ruminococcus* | -0.46 | 0.02 |
| 45 | *Paraprevotella* | -0.20 | 0.005 |
| 47 | *Alphaproteobacteria class (unclassified)* | +0.16 | < 0.01 |
| 48 | *Alphaproteobacteria class (unclassified)* | +2.37 | 0.003 |
| 50 | *Bacteroides* | +012 | 0.04 |
| 58 | *Bacteroidales order (unclassified)* | +0.001 | 0.05 |
| 60 | *Faecalibacterium* | -0.29 | 0.05 |
| 65 | *Lachnospiraceae family (unclassified)* | +0.07 | 0.01 |
| 67 | *Prevotella* | -0.03 | 0.04 |
| 74 | *Bacteroidales order (unclassified)* | +0.03 | 0.007 |
| 91 | *Clostridium Cluster IV (unclassified)* | -0.001 | 0.01 |
| 115 | *Clostridiales family (unclassified)* | +0.008 | 0.007 |
| 210 | *Bacteroidetes phylum (unclassified)* | -0.001 | 0.02 |
| 238 | *Lachnospiraceae family (unclassified)* | +0.009 | 0.02 |
|  |  |  |  |
| **Table 3 (continued)**  **Operational Taxonomic Units (OTU’s) significantly different between Mycoprotein and Meat** | | | |
| No. Operational Taxonomic Unit (OTU) | Bacterial Taxonomic Classification | Difference in change in relative abundance between diets^2^ | *P^3^* |
| 3 | *Faecalibacterium* | -4.03 | 0.02 |
| 11 | *Roseburia* | +2.22 | 0.001 |
| 13 | *Bacteroides* | -3.00 | 0.01 |
| 15 | *Sutterella* | +1.35 | 0.03 |
| 20 | *Prevotella* | -2.86 | < 0.001 |
| 28 | *Lachnospiraceae family (unclassified)* | +1.25 | < 0.001 |
| 32 | *Oscillibacter* | -0.66 | 0.004 |
| 34 | *Akkermansia* | +1.66 | 0.02 |
| 35 | *Barnesiella* | +0.12 | 0.02 |
| 45 | *Parabacteroides* | +0.64 | 0.001 |
| 48 | *Alphaproteobacteria class (unclassified)* | -0.23 | 0.001 |
| 49 | *Alphaproteobacteria class (unclassified)* | -1.66 | 0.05 |
| 51 | *Bacteroides* | +0.58 | 0.02 |
| 66 | *Lachnospiraceae family (unclassified)* | -0.07 | 0.007 |
| 75 | *Bacteroidales order (unclassified)* | -0.02 | 0.03 |
| 92 | *Clostridium Cluster IV (unclassified)* | +0.001 | 0.001 |
| 108 | *Prevotella* | -0.02 | 0.008 |
| 116 | *Clostridiales order (unclassified)* | -0.008 | 0.007 |
| 156 | *Clostridiales order (unclassified)* | +0.02 | 0.02 |
| 239 | *Lachnospiraceae family (unclassified)* | -0.009 | 0.04 |
|  |  |  |  |
| ***1*** Results presented as least square mean change from baseline for the study phases.  **2** Difference between diets calculated by subtracting change following Mycoprotein by change following Meat.  **3** *P* values were calculated for changes within each study phase and differences between study phases using generalised mixed effects models. Differences regarded as significant at a *P* < 0.05. | | | |

**Figure 2. Comparison of faecal metabolome between Meat and Mycoprotein.
A)** Partial least square discriminant analysis (PLS-DA) of faecal metabolites profiles by the primary and secondary components, at study phase completion. The first two components accounted for ~27.4 % of variability (9.6% and 17.8% for the first and second components respectively). **B) and C)** Metabolic pathway enrichment using faecal metabolites considered a VIP >1.5 between **B)** Meat and **C)** Mycoprotein.


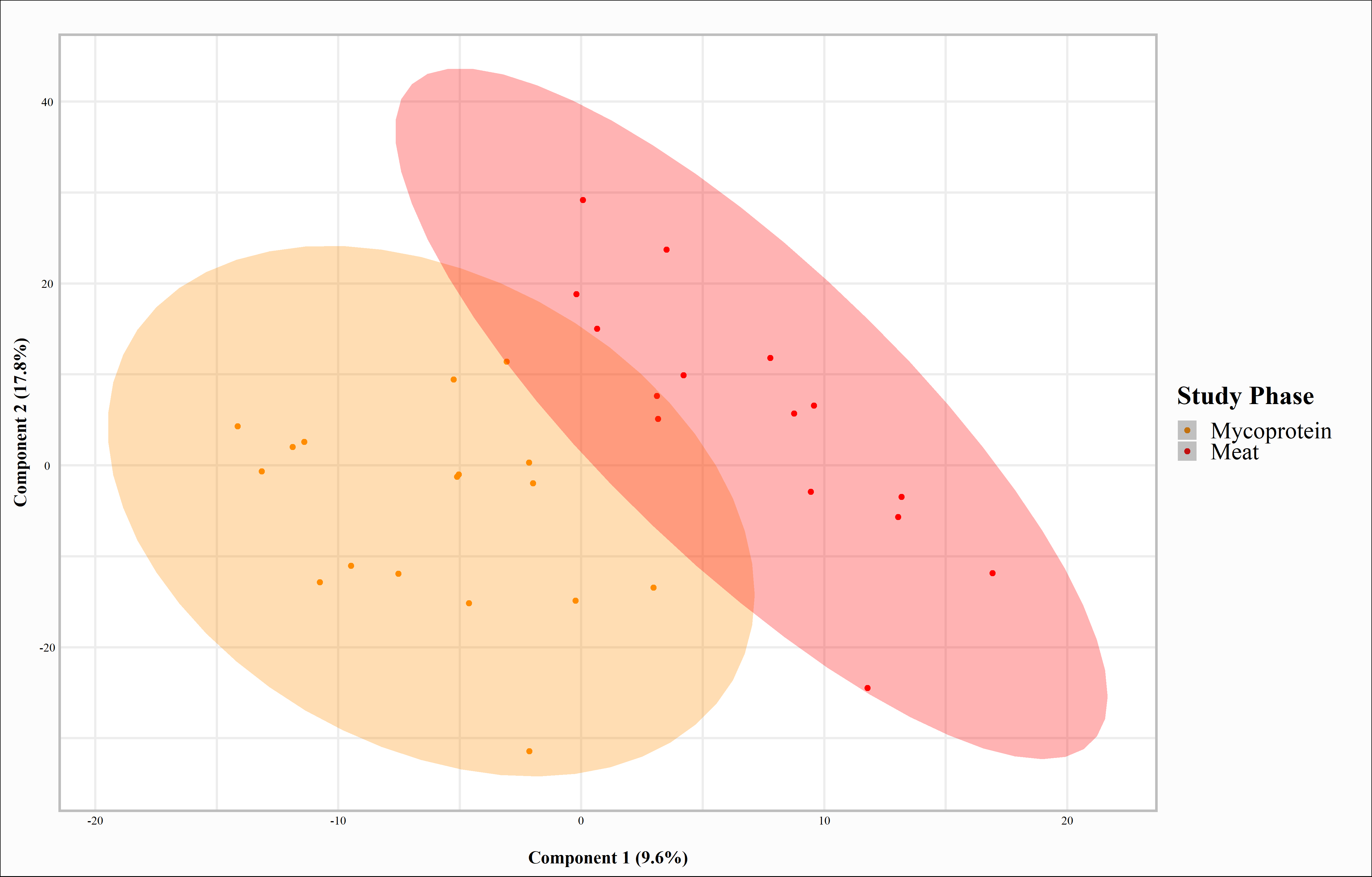


A

C

B


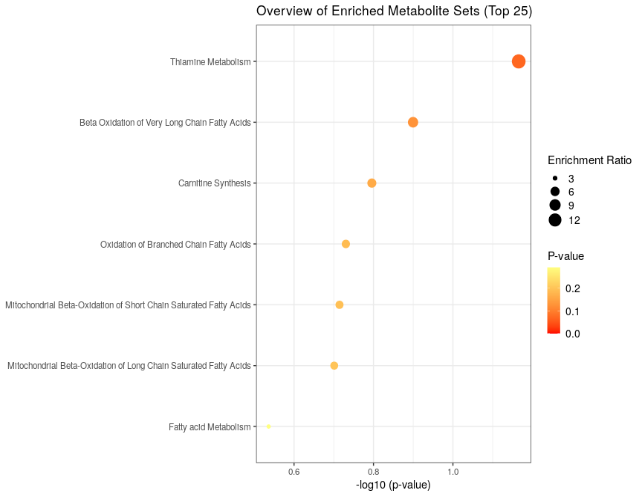

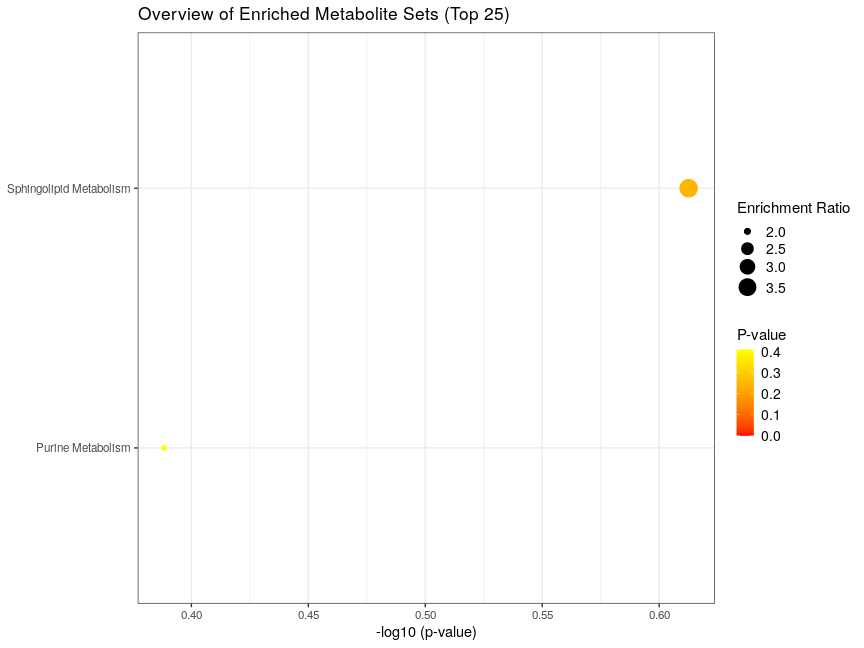


| **Table 4. Faecal metabolites considered a VIP >1.5 between Meat and Mycoprotein^1,2^** | | |
| --- | --- | --- |
| **Metabolite** | **VIP** | **Fold Change (Mycoprotein/Meat)** |
| C22 H38 N4 O7 | 6.74 | 4.50 |
| C7 H17 N O2.1 | 6.39 | 4.26 |
| C15 H26 N2 O5 | 5.14 | 3.43 |
| leucyl-4-hydroxyproline | 5.10 | 3.40 |
| C14 H24 N2 O5 | 5.01 | 3.34 |
| C16 H29 N O3 | 3.87 | 2.58 |
| 4-Hydroxyprolylleucine | 3.52 | -2.34 |
| C10 H29 N6 O4 P | 3.43 | 2.29 |
| C25 H36 N8 O4 | 3.33 | 2.22 |
| N-[(2Z)-2-Hydroxy-2-octenoyl]glycine | 3.16 | 2.11 |
| Ile-val | 3.00 | -2.00 |
| Asparaginyl-4-hydroxyproline | 2.91 | -1.94 |
| C8 H16 N2 O.1 | 2.87 | -1.92 |
| C32 H60 N2 O14 | 2.84 | 1.90 |
| C13 H26 N9 O7 P | 2.82 | 1.88 |
| C32 H44 N2 O10 | 2.75 | 1.83 |
| N~6~,N~6~-Dimethyllysine | 2.70 | -1.80 |
| dinotefuran | 2.65 | -1.77 |
| C7 H12 N4 O2 | 2.57 | -1.72 |
| C9 H19 N2 O6 P | 2.51 | 1.67 |
| Isoputreanine | 2.42 | -1.62 |
| Glycylglutamine | 2.41 | -1.60 |
| L(+)-Ornithine | 2.33 | 1.55 |
| Gamma-Aminobutyric acid (GABA) | 2.26 | 1.51 |
| C16 H34 N2 O4 | 2.15 | -1.43 |
| C7 H14 N2 O | 2.14 | -1.43 |
| 4-[(3-Acetamidopropyl)amino]butanoic acid | 2.14 | -1.42 |
| 1,3-Dipropylxanthine | 2.13 | 1.42 |
| C12 H26 N2 O4 | 2.08 | -1.39 |
| Butenylcarnitine | 2.06 | 1.37 |
| C10 H15 N O3 S | 2.04 | 1.36 |
| C33 H54 N8 O9 P2 | 2.02 | 1.35 |
| **Table 4 (continued)** | | |
| P-DMEA | 2.00 | -1.34 |
| (2S)-2-Amino-8-hydroxyoctanoic acid.1 | 1.98 | -1.32 |
| C6 H7 N3 | 1.93 | 1.29 |
| Sorbic acid | 1.90 | 1.27 |
| Valyl-4-hydroxyproline | 1.88 | -1.25 |
| Thiamine | 1.88 | 1.25 |
| 4-Methyl-5-thiazoleethanol | 1.86 | 1.24 |
| ISOXYL.1 | 1.85 | 1.24 |
| Allysine | 1.81 | -1.21 |
| C10 H18 N4 O3 | 1.80 | -1.20 |
| porfiromycin | 1.79 | -1.19 |
| C89 H135 N6 O18 P | 1.75 | 1.17 |
| C28 H29 N5 O6 | 1.75 | 1.17 |
| C20 H46 N5 O7 P | 1.67 | -1.11 |
| C9 H18 N4 O | 1.65 | -1.10 |
| C89 H149 N8 O15 P3 S | 1.63 | 1.09 |
| C10 H16 N6 O2 | 1.62 | -1.08 |
| Homocarnosine | 1.58 | -1.05 |
| Fusicoccin H | 1.57 | -1.05 |
| DL-Atenolol | 1.54 | 1.03 |
| V594 | 1.54 | 1.03 |
| C16 H27 N3 O7 | 1.53 | -1.02 |
| C35 H71 N O4 | 1.52 | 1.01 |
| L-gamma-Glutamyl-L-leucine | 1.50 | -1.00 |
| ^1^VIP threshold set at >1.5.  ^2^Unidentified metabolites presented as chemical formula (i.e., C8 H16 N2 0)  Positive fold change indicates greater Mycoprotein, negative fold change indicates greater Meat.  VIP, Variable importance in projection | | |

**Figure 3. Comparison of urine metabolome between Meat and Mycoprotein.
A)** Partial least square discriminant analysis (PLS-DA) of urine metabolites profiles by the primary and secondary components, at study phase completion. The first two components accounted for ~15.27% of the variability (7.73% and 7.54% for the first and second components, respectively). **B) and C)** Metabolic pathway enrichment using urine metabolites considered a VIP >1.5 for **B)** Meat and **C)** Mycoprotein.


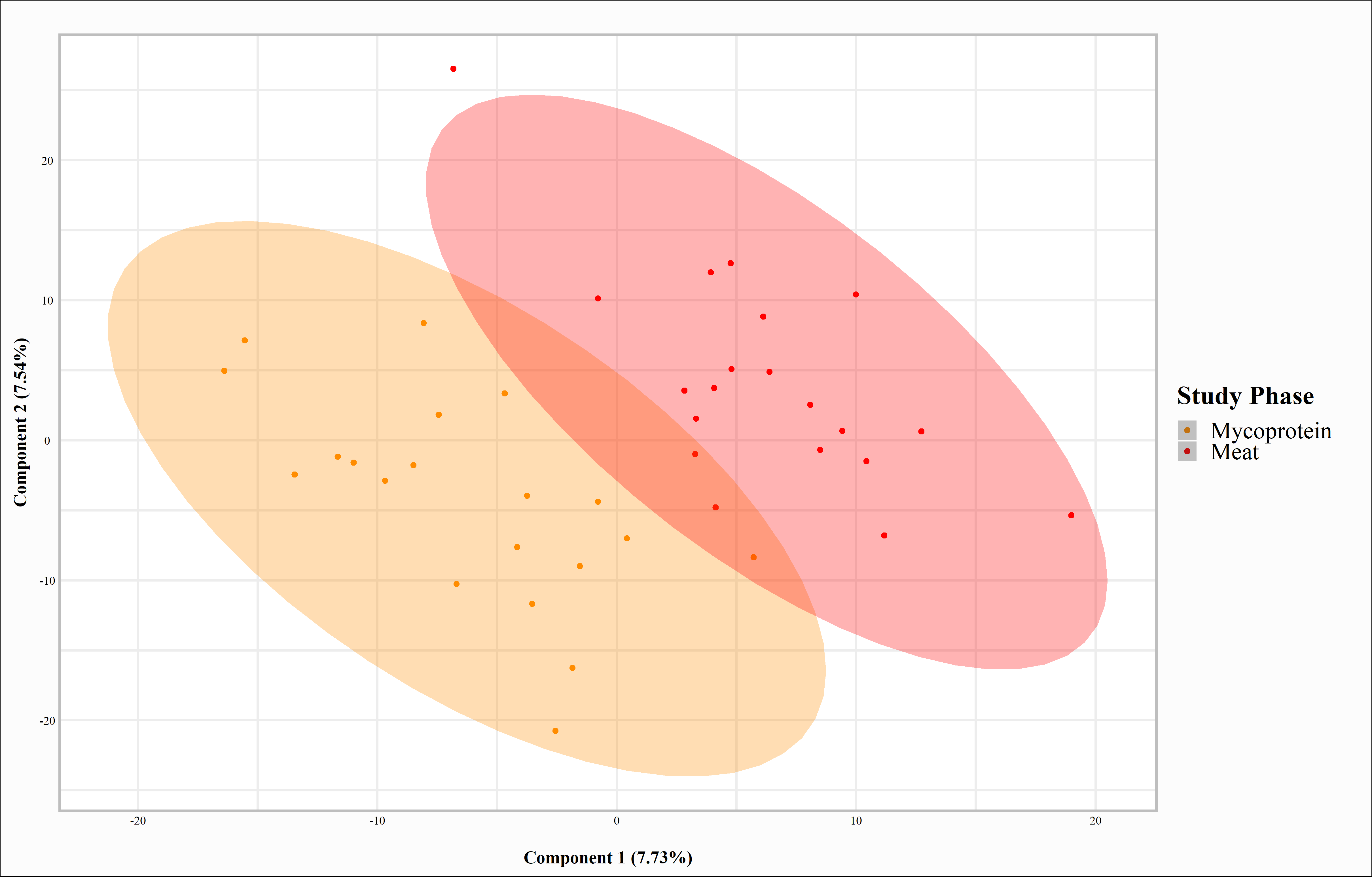


A

C

B


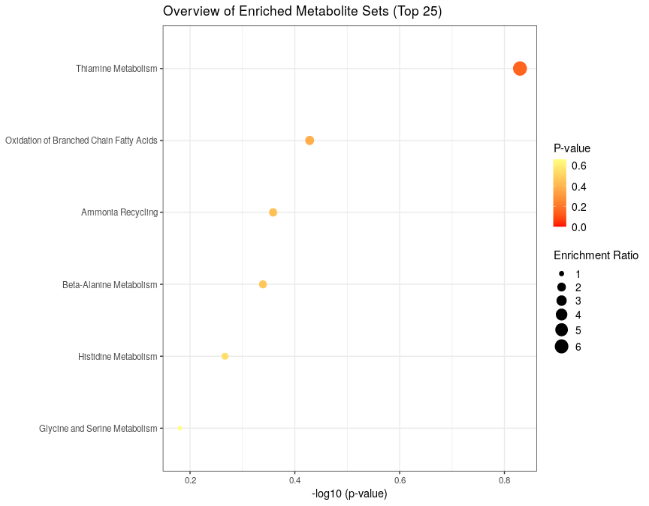

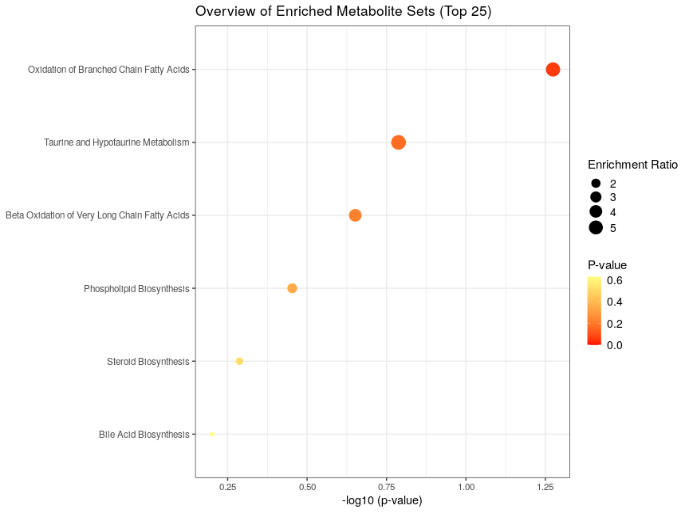


| **Table 5. Urine metabolites considered a VIP >1.5 between Meat and Mycoprotein^1,2^** | | |
| --- | --- | --- |
| **Metabolite** | **VIP** | **Fold Change (Mycoprotein/Meat)** |
| balenine.1 | 5.65 | -3.65 |
| C11 H18 N2 O6 | 4.52 | 2.92 |
| C9 H15 N3 O | 4.11 | -2.66 |
| C8 H14 N6 O7 | 4.08 | 2.64 |
| C14 H30 N2 O6 | 3.99 | -2.58 |
| C10 H21 N5 O3 | 3.82 | -2.47 |
| C17 H31 N O3 | 3.76 | 2.43 |
| l-Histidinal | 3.35 | -2.16 |
| val-his | 3.20 | -2.07 |
| N-[(2Z)-2-Hydroxy-2-octenoyl]glycine | 2.99 | 1.94 |
| C12 H25 O6 P3 | 2.95 | -1.90 |
| C13 H20 N2 O5 | 2.87 | -1.85 |
| C7 H13 N3 O | 2.85 | 1.84 |
| Miglitol | 2.70 | -1.75 |
| C10 H19 N5 O2 | 2.66 | -1.72 |
| C34 H44 N8 O2 | 2.62 | -1.70 |
| C30 H51 N4 O2 P3 | 2.61 | -1.69 |
| C29 H42 N10 O4 | 2.57 | -1.66 |
| C19 H26 O9 | 2.57 | 1.66 |
| Propionylcarnitine | 2.55 | -1.65 |
| gamma-thiomethyl glutamate | 2.51 | 1.62 |
| C10 H4 N3 O3 P | 2.51 | -1.62 |
| Ibuprofen metabolite B | 2.48 | 1.60 |
| (3S,6S)-3-(Hydroxymethyl)-6-{4-[(3-methyl-2-buten-1-yl)oxy]benzyl-2,5-piperazinedione | 2.46 | -1.59 |
| TDIQ | 2.37 | -1.53 |
| 2-(6,10-Dimethyl-8-oxospiro[4.5]dec-6-en-2-yl)-2-hydroxypropyl hexopyranoside | 2.36 | 1.52 |
| 9-Nitrooleate | 2.32 | -1.50 |
| N(alpha)-gamma-L-glutamylhistamine | 2.26 | -1.46 |
| (5E)-7-methylidene-10-oxo-4-(propan-2-yl)undec-5-enoic acid | 2.20 | 1.42 |
| **Table 5 (continued)** | | |
| C22 H33 N5 O4 | 2.19 | 1.42 |
| C10 H18 N4 O3 | 2.19 | -1.41 |
| C29 H47 N O8 S | 2.12 | 1.37 |
| Allysine.1 | 2.11 | -1.36 |
| C8 H13 N O4 S.1 | 2.07 | 1.34 |
| C10 H13 N O5 S.1 | 2.05 | 1.33 |
| Acetyl-Î²-methylcholine | 2.03 | -1.31 |
| Acetylcholine | 2.03 | -1.31 |
| Xanthotoxol | 2.01 | 1.30 |
| 3-Methylglutarylcarnitine | 2.00 | -1.29 |
| Mevalonic acid | 1.99 | 1.29 |
| C6 H8 O7 S.1 | 1.95 | -1.26 |
| Sorbic acid | 1.92 | 1.24 |
| Acronycidine | 1.92 | -1.24 |
| C10 H27 N4 O3 P3 | 1.90 | 1.23 |
| C11 H20 N2 O7 S3 | 1.88 | 1.21 |
| C21 H31 N O9 | 1.87 | -1.21 |
| 5-(2,3-Dihydroxy-3-methylbutyl)-4-[(3,3-dimethyl-2-oxiranyl)acetyl]-3,4-dihydroxy-2-(3-methylbutanoyl)-2-cyclopenten-1-one | 1.85 | 1.20 |
| DL-Î²-Leucine | 1.81 | -1.17 |
| C8 H12 N2 O5 S | 1.81 | -1.17 |
| C12 H21 N3 O4 S | 1.81 | 1.17 |
| methoxyresorcinol | 1.81 | -1.17 |
| C5 H10 N8 O S2 | 1.78 | 1.15 |
| C15 H25 N7 O6 | 1.77 | 1.15 |
| C10 H25 N6 O9 P S3.1 | 1.77 | -1.14 |
| C16 H34 N O7 P3 | 1.76 | 1.14 |
| C14 H20 N2 O8 | 1.76 | -1.14 |
| Taurine | 1.75 | -1.13 |
| C16 H19 N O6 S.1 | 1.74 | -1.12 |
| Taurine.1 | 1.74 | -1.12 |
| N-[2-Hydroxy-4-(sulfooxy)phenyl]acetamide | 1.72 | -1.11 |
| C14 H18 N2 O3 S2 | 1.70 | 1.10 |
| **Table 5 (continued)** | | |
| C7 H16 N8 O3 S3 | 1.70 | -1.10 |
| C22 H19 N4 O3 P S | 1.70 | 1.10 |
| ibufenac.1 | 1.70 | 1.10 |
| 2-methoxyacetaminophen sulfate | 1.69 | -1.10 |
| paracetamol sulfate | 1.69 | -1.09 |
| Arbusculin A | 1.68 | 1.09 |
| 5-Methylangelicin | 1.67 | -1.08 |
| C6 H17 N4 O8 P | 1.67 | 1.08 |
| C22 H35 N O8 | 1.67 | -1.08 |
| C6 H14 N5 O7 P S | 1.65 | -1.07 |
| Arsenobetaine | 1.65 | -1.06 |
| 3-[2-[(E)-[3-(2-carboxyethyl)-5-[(4-ethyl-3-methyl-5-oxo-pyrrolidin-2-yl)methyl]-4-methyl-pyrrol-2-ylidene]methyl]-5-[(3-ethyl-4-methyl-5-oxo-pyrrolidin-2-yl)methyl]-4-methyl-1H-pyrrol-3-yl]propanoic acid | 1.65 | -1.06 |
| N-Acetyl-L-cysteine | 1.63 | 1.05 |
| C24 H26 N6 O6 | 1.61 | 1.04 |
| Succinylacetone | 1.59 | 1.03 |
| C5 H4 O7 S | 1.56 | 1.01 |
| L-Anserine | 1.55 | -1.00 |
| C6 H12 N3 O3 P.1 | 1.54 | 1.00 |
| C12 H24 N5 O6 P S | 1.54 | -0.99 |
| Roxatidine | 1.54 | -0.99 |
| N-Acetylleucylleucine | 1.50 | 0.97 |
| ^1^VIP threshold set at >1.5.  ^2^Unidentified metabolites presented as chemical formula (i.e., C8 H16 N2 0)  Positive fold change indicates greater Mycoprotein, negative fold change indicates greater Meat.  VIP, Variable importance in projection | | |
